# Dopant-Induced Hexagonal to Orthorhombic Phase Transition in Fe_2–*x*_Mo_*x*_P Nanorods and Its Influence on the Electrocatalytic Hydrogen Evolution Reaction

**DOI:** 10.1021/acs.chemmater.4c03479

**Published:** 2025-04-28

**Authors:** Jordon Baker, Danyang Wang, Md Kawsar Alam, Ka Un Lao, Indika U. Arachchige

**Affiliations:** Department of Chemistry, Virginia Commonwealth University, Richmond, Virginia 23284-2006, United States

## Abstract

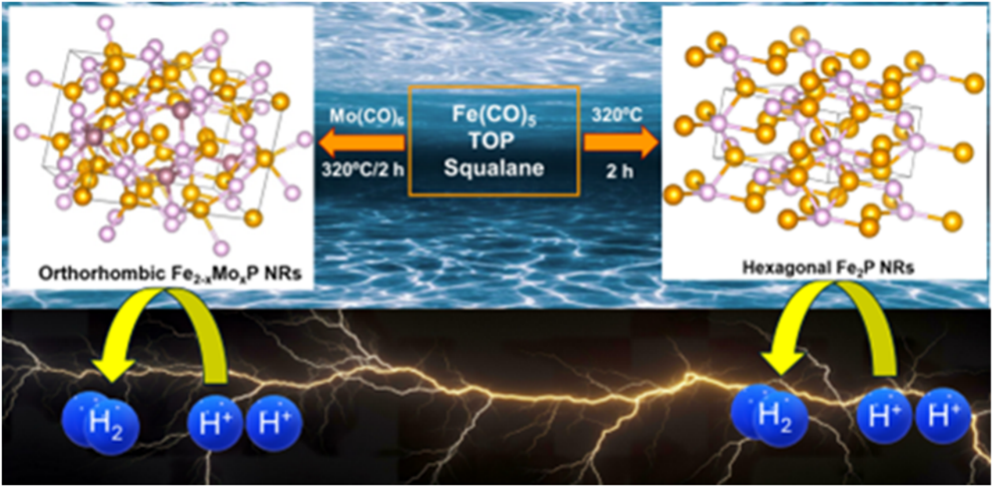

Electrochemical water splitting represents a sustainable method for producing molecular hydrogen, a promising clean energy alternative to fossil fuels. Iron phosphides have emerged as earth-abundant catalysts for the hydrogen evolution reaction (HER), where performance can be enhanced by admixing synergetic metals to produce bimetallic catalysts. Herein, we report a theoretical and experimental study that reveals the influence of dopant-induced hexagonal to orthorhombic phase transition on the catalytic activity and stability of Fe_2_P nanorods (NRs) for HER. Among eight metal dopants computationally studied, Mo has been identified as the most promising dopant owing to its optimum hydrogen binding free energy (Δ*G*_H_) on the Fe_2_P (210) surface. Accordingly, hexagonal and orthorhombic Fe_2–*x*_Mo_*x*_P NRs (*x* = 0–14%) with average lengths and widths ranging from 50.9 ± 22.1 to 92.4 ± 43.8 nm and 3.8 ± 1.0 to 6.3 ± 1.8 nm, respectively, were colloidally synthesized to investigate the structure- and composition-dependent HER activity. Upon incorporation of Mo, the underlying hexagonal Fe_2_P phase transformed into orthorhombic Fe_2–*x*_Mo_*x*_P when *x* ≥ 0.11 (5.41%). The admixture of Mo caused variations in the surface chemistry, leading to a significant decrease in Fe^δ+^ and P^δ−^ charges. The HER performance was observed to be both phase- and composition-dependent with mixed-phase Fe_2–*x*_Mo_*x*_P NRs (*x* = 0.03, 0.06, and 0.09) exhibiting superior catalytic activity and overpotentials (η_–10_) of 298, 267, and 222 mV, respectively at a current density (*j*) of −10 mA/cm^2^ compared to hexagonal Fe_2_P (η_–10_ = 378 mV) and orthorhombic Fe_2–*x*_Mo_*x*_P (η_–10_ = 331–459 mV for *x* = 0.12–0.28) catalysts. The highest HER performance was achieved for Fe_1.91_Mo_0.09_P NRs with a dopant composition of 4.58%, consistent with composition-dependent Δ*G*_H_ calculations. Although all compositions displayed a Volmer–Heyrovsky HER mechanism, the admixture of Mo improved the HER kinetics, producing the lowest Tafel slope (167.08 mV/dec) for Fe_1.91_Mo_0.09_P NRs. The incorporation of Mo improves the charge transfer resistance and preserves the stability of hexagonal and orthorhombic NRs in alkali environments with a negligible increase in η_–10_ after 10 h of HER. This study advances the understanding of dopant-induced crystal structure transitions and paves the way for efficient and stable catalytic material design.

## Introduction

For decades, society has depended on fossil fuels and nonrenewable energy sources to power motor vehicles and generate electricity, increasing greenhouse gas emissions and global warming. This issue, which has been widely ignored since the advent of the industrial revolution, has prompted us to reassess the use of fossil fuels and search for alternative, sustainable, and renewable energy sources. Many carbon-neutral energy technologies are being studied, including solar, wind, hydroelectric, geothermal, and hydrogen.^1,2^ Among those, hydrogen is the most promising source, which carries the highest energy density per unit mass of any fuel used today.^3,4^ Producing water when it burns, hydrogen is also the cleanest of all energies. Because it is lighter than air, hydrogen exists primarily in nature as a compound and must be produced.^5^ Electrochemical water splitting presents an exciting opportunity for hydrogen generation. Currently, noble metals such as Pt, Rh, Ir, and Ru are being used as high-performance electrocatalysts for water splitting; nonetheless, their high cost limits widespread use.^6^ The hydrogen absorption free energy (Δ*G*_H_) of transition elements follows a volcano trend, showing an optimum Δ*E*_M–H_ to absorb reactants and rapidly desorb products.^7,8^ A Δ*G*_H_ closer to zero is desired to rapidly generate H_2_ without irreversible adhesion to the catalyst surface. Noble metal catalysts, at the top of the volcano plot, show an optimum Δ*E*_M–H_ sufficient to absorb reactants and rapidly desorb products. In contrast, earth-abundant transition metals (e.g., Ni, Mo, Fe, Cu, V, and Co) show significant deviations from the optimum Δ*E*_M–H_, causing either no adsorption or irreversible absorption of reactants and products. This deficiency has been attributed to the limited tunability of surface affinity and adsorption strength of metal–hydrogen intermediates for monometallic phosphides.^9,10^ However, homogeneous admixing of synergistic transition metals and/or incorporation of nonmetal elements (e.g., chalcogenides, nitrites, and phosphides) can be explored to optimize Δ*G*_H_ and enhance the HER activity and stability of earth-abundant catalysts.^11−16^

Among transition metal phosphides, iron phosphides have gained interest in a range of applications that vary based on the stoichiometry and crystal structure. Out of the five stable structures of Fe_3_P, Fe_2_P, FeP, FeP_2_, and FeP_4_,^17−21^ Fe_3_P exhibits ferromagnetic properties,^22^ whereas FeP_2_ is a small bandgap semiconductor.^23^ On the contrary, FeP, Fe_2_P, FeP_2_, and FeP_4_ are promising catalysts for HER and oxygen (OER) evolution reactions, nitrate reduction, and ammonia synthesis.^24−31^ Specifically, Fe_2_P has been identified as a promising HER catalyst because of its low overpotential, fast kinetics, and high stability in acidic and alkaline electrolytes.^32,33^ Fe_2_P has garnered additional interest due to its unique, naturally occurring nanorod (NR) morphology. Cho and co-workers^34^ reported the colloidal synthesis of cubic Fe nanoparticles (NPs), hexagonal Fe_2_P NRs, and orthorhombic FeP NRs and probed their HER activity in acid electrolytes. An improved understanding of the material’s properties, including crystal phase and morphology, was gained by manipulating the synthesis parameters.^35^ Additionally, the catalytic studies reported the lowest overpotentials (η_–10_) for FeP NRs, compared to Fe NPs and Fe_2_P NRs, indicating a higher HER activity. However, when compared with Pt/C, all iron phosphide catalysts showed lower HER activity with significantly higher η_–10_ values. On the other hand, several studies on transition-metal-doped iron phosphides have also been reported. These reports demonstrate enhanced HER activity of Fe_2_P and FeP nanostructures doped with Co,^33,36^ Ni,^32^ Mn,^37^ V,^38^ Nb,^39,40^ and Ag^41^ in acidic and alkaline electrolytes. Specifically, the introduction of V^38^ into the Fe_2_P@Co_3_(PO_4_)_2_ composite nanosheets has been reported to decrease (42.8%) η_–10_ dramatically in alkaline solutions. Interestingly, when Ni was employed as a dopant for Fe_2_P nanocrystals (NCs),^32^ a composition-dependent variation in HER activity has been reported with Fe_0.5_Ni_1.5_P NCs demonstrating the highest activity (η_–50_ = 163 mV) compared to Fe_2_P (η_–50_ = 217 mV), Ni_2_P (η_–50_ = 170 mV), and Fe_1.0_Ni_1.0_P (η_–50_ = 178 mV) NCs. Although a few transition metal dopants have been shown to promote the HER activity of hexagonal Fe_2_P, a systematic investigation of the HER performance of Fe_2–*x*_Mo_*x*_P nanostructures, to the best of our knowledge, has not been reported.

Herein, density functional theory (DFT) calculations were utilized to explore the influence of transition metal dopants on the Δ*G*_H_ of hexagonal and orthorhombic Fe_2_P. Among earth-abundant dopants computationally investigated in this study, Mo emerged as the most promising HER dopant for Fe_2_P, decreasing the overall |Δ*G*_H_|, |Δ*G*_H1_| + |Δ*G*_H2_|, to 0.26 eV. In contrast, none of the earth-abundant dopants showed a notable impact on the Δ*G*_H_ of the hexagonal Fe_2_P. To the best of our knowledge, Mo has not been investigated as a dopant in experimental HER studies of Fe_2_P nanostructures, nor are there comprehensive theoretical predictions on the HER activity of Fe_2–*x*_Mo_*x*_P catalysts. Motivated by these gaps, along with the known HER activity of Mo-based compounds such as MoP, their synthetic feasibility, and industrial relevance, we were prompted to explore Mo as a promising dopant for enhancing the HER performance of Fe_2_P NRs. The incorporation of Mo decreased the stability of hexagonal Fe_2_P and caused a phase transition to orthorhombic Fe_2_P. Accordingly, a series of Fe_2–*x*_Mo_*x*_P NRs (*x* = 0–0.28) with hexagonal and orthorhombic structures were colloidally synthesized to probe the HER activity as a function of composition. As-synthesized NRs retained the rod morphology of hexagonal Fe_2_P; however, a phase transition to orthorhombic Fe_2_P structure was experimentally observed when *x* ≥ 0.11 (5.41%). Surface analysis of Fe_2–*x*_Mo_*x*_P NRs revealed significant variations in surface chemistry with a decrease in Fe^δ+^ and P^δ−^ charges compared to hexagonal Fe_2_P. The Fe_2–*x*_Mo_*x*_P NRs showed enhanced electrocatalytic activity with a decrease in η_–10_ from 298 to 222 mV for *x* = 0.03–0.09 compositions, compared to 378 mV realized for Fe_2_P. The highest HER activity was achieved for Fe_1.91_Mo_0.09_P (222 mV) composition, consistent with composition-dependent Δ*G*_H_ calculations. The homogeneous admixture of Mo improves the HER kinetics, increases the charge transfer resistance, and maintains the corrosion tolerance of Fe_2_P NRs in alkaline electrolytes. To the best of our knowledge, this is the first study of Mo-doped Fe_2_P NRs that demonstrates a structure transition upon doping with heteroatoms and investigates the influence of crystal phases and compositions on HER activity and stability.

## Experimental Section

### Materials

Iron pentacarbonyl (Fe(CO)_5_, 99.99%) was purchased from Sigma-Aldrich and was stored in a nitrogen glovebox at 0 °C. Trioctylphosphine (TOP, 97%) was purchased from Strem Chemicals. Squalane (98%), molybdenum hexacarbonyl (Mo(CO)_6_, 98%), oleylamine (OLA, 70%), toluene, and ethanol were purchased from Fisher. Carbon-coated 200-mesh copper grids were purchased from SPI Supplies. Titanium foil (thickness 0.25 mm; 99.7%) and platinum on graphitized carbon (Pt/C, 10 wt %) were purchased from Sigma-Aldrich. Graphite rods (6.15 × 102 mm^2^, 99.99995%) were purchased from Alfa Aesar. A Hg/HgO reference electrode filled with a 1 M NaOH solution was purchased from CH Instruments. Pelco colloidal silver paint was purchased from Ted Pella. Henkel Loctite EA-E00NS epoxy adhesive and PTFE-insulated silver-plated copper wires were purchased from McMaster-Carr. Squalane and OLA were dried under vacuum for 3 h prior to use and stored in a N_2_ glovebox. Toluene was dried over Na, and ethanol was dried over CaO and molecular sieves. Both solvents were distilled under N_2_ before use.

### Synthesis of Fe_2_P NRs

The synthesis was derived from the literature.^35^ In a glovebox, 5.2 mmol of Fe(CO)_5_ were added to a 100 mL three-neck round-bottom flask along with 5 mL of TOP and 10 mL of OLA. This setup was attached to a Schlenk line and the solution was degassed under vacuum at 120 °C for 30 min. Then, nitrogen gas was flowed into the reaction flask, and steady reflux through a condenser was introduced. Before the temperature was increased to 320 °C, the nitrogen flow was removed once the bubbler was properly attached to prevent iron nitride formation. Once 320 °C was achieved, the reaction was held at this temperature for 1 h. Then, the flask was cooled to room temperature, and the product was isolated with a mixture of toluene and ethanol and centrifuged at 6000 rpm for 10 min. This purification process was repeated three times, and the final product was dried under vacuum.

### Synthesis of Fe_2–*x*_Mo_*x*_P NRs

In a glovebox, appropriate amounts of Fe(CO)_5_ and Mo(CO)_6_ were mixed in a three-necked 100 mL flask along with 5 mL of TOP and 10 mL of Squalane. This flask was attached to a Schlenk line using a condenser, and the solution was degassed under vacuum at 120 °C for 30 min. Afterward, the reaction was continued without further nitrogen flow to prevent the formation of iron nitride. Next, cold water was refluxed in the condenser, the temperature was increased to 320 °C, and the reaction was continued at 320 °C for 2 h. Then, the flask was cooled to room temperature using compressed air, and the Fe_2–*x*_Mo_*x*_P NRs were isolated similarly to Fe_2_P NRs synthesis.

### Fabrication of Working Electrodes

Working electrodes were fabricated with Fe_2_P and Fe_2–*x*_Mo_*x*_P NRs and commercial Pt/C (10 wt %) catalyst on Ti substrates. Ti foils were cut into 0.5 cm × 0.4 cm pieces, subjected to sonication in a solution of acetone and ethanol (1:1, v/v) for 20 min, and rinsed off with deionized water. Then, the foils were immersed in a 1 M HCl and 30% H_2_O_2_ (1:1 v/v) mixture for an additional 30 min, rinsed with deionized water, and left to dry overnight. The catalyst ink solution was prepared by mixing 4 mg of NRs or 10% Pt/C with 368 μL isopropanol, 368 μL of ultrapure water, and 48.1 μL of purified Nafion. This mixture was sonicated for 1 h in an ice water bath. Nafion was neutralized by mixing 2 mL of Nafion and 1 mL of 0.1 M KOH, followed by stirring overnight. 10 μL aliquots of catalyst ink were drop-cast onto Ti foil until weight loading reached ∼300 μg. Each aliquot was dried in air before the next aliquot was drop-cast. To improve the adhesion of NRs and to remove residual organic ligands, the catalyst-coated Ti foils were annealed in a tube furnace at 450 °C for 2 h under 5% H_2_/Ar. Then, Ag paint was used to attach catalyst-coated Ti foil to a Ag-plated Cu wire and establish a proper ohmic contact. After the Ag paint was dried, a two-part epoxy was used to cover and insulate the Ag paint and Cu wire while leaving the catalytic area (0.20 cm^2^) exposed for HER studies. The epoxy was left to dry for 24 h before the electrocatalytic experiments.

### Electrochemical Measurements

The HER activity of Fe_2_P and Fe_2–*x*_Mo_*x*_P NRs and commercial Pt/C catalysts was investigated using a CHI 760E potentiostat. All experiments were conducted in a nitrogen-saturated 1 M KOH solution at room temperature and pressure using a standard three-electrode electrochemical cell. A graphite rod was used as a counter electrode, Hg/HgO (1 M NaOH) was used as the reference electrode, and catalyst-coated Ti foil served as the working electrode. Prior to data collection, N_2_ gas was purged into the system for 2 h to remove atmospheric gases and maintain an inert environment throughout the experiment. The potentials were reported with respect to the scale of the reversible hydrogen electrode (RHE) potential by using the conversion formula: *E*_RHE_ = *E*_exp_ + *E*_Hg/HgO_^0^ + 0.05916 pH. The current densities (*j*, mA/cm^2^) of catalysts were calculated by dividing the experimental current by the geometrical area of the electrode surface (0.2 cm^2^). Cyclic voltammetry (CV) was conducted by sweeping the potential from 0.2 to 0.1 V (vs RHE) at different scan rates to electrochemically clean and activate the catalyst. Linear sweep voltammetry (LSV) was used to record polarization curves by scanning the potential from 0.2004 to –0.8 V (vs RHE) at a scan rate of 5 mV/s. Electrochemical Impedance Spectroscopy (EIS) measurements were conducted in 1 M KOH at a HER overpotential of −250 mV vs RHE with a frequency range of 10^5^ to 0.1 Hz. Chronopotentiometry analysis was performed at current densities (*j*) of −10 mA/cm^2^ for 10 h in N_2_-saturated 1 M KOH for selected catalysts.

### Physical Characterization

Powder X-ray diffraction (PXRD) patterns were recorded using an Empyrean Multipurpose X-ray diffractometer equipped with Cu Kα (λ = 1.5418 Å) radiation operating at 45 kV and 40 mA. This diffractometer was precalibrated with silicon standard. Diffraction patterns were analyzed by using HighScore Plus software. Low (LR)- and high (HR)-resolution transmission electron microscopy (TEM) images, scanning TEM (STEM) images, and elemental maps were recorded by using a JEM-F200 Cold FEG electron microscope operating at an accelerating voltage of 200 kV and equipped with an energy-dispersive X-ray (EDAX) analyzer. TEM samples were prepared by drop-casting a 10 μL aliquot of a dilute NC-toluene solution onto 200-mesh carbon-coated grids, followed by evaporation of the solvent. X-ray photoelectron spectra (XPS) were recorded by using a PHI VersaProbe III Scanning XPS Microprobe. Samples were annealed at 450 °C for 2 h under 5% H_2_:Ar prior to XPS analysis. Regional scans were completed using a pass energy of 26.00 eV with 20 ms per step, and the number of sweeps was dependent upon the signal intensity of each element. Elemental compositions were determined from Hitachi Ultra High-Resolution Analytical FE-SEM SU-70, operating at 15 kV with an energy-dispersive spectroscopy (EDS) X-ray analyzer. The average elemental compositions were determined by averaging the atomic percent of each element across 5 individual spots per sample.

### Computational Studies

To identify the optimal dopant and dopant concentration for enhancing the HER activity of Fe_2_P, eight transition metal dopants were studied: Co, Cr, Cu, Mn, Mo, Ni, V, and Ti. The electrocatalytic activity was evaluated based on the computation of the Gibbs free energy for HER, expressed as Δ*G*_H_ = Δ*E* + Δ*E*_ZPE_ – *T*Δ*S*. Here, Δ*E* represents the electronic adsorption energy derived from DFT calculations, Δ*E*_ZPE_ denotes zero-point energy, T denotes temperature, and Δ*S* embodies entropy contributions. The Δ*G*_H_ binding interactions are a critical determinant of intrinsic HER activity, with the optimum catalyst characterized by a Δ*G*_H_ closer to 0 eV consistent with the Sabatier principle. An empirical adjustment factor of 0.24 eV was applied to account for entropic and zero-point energy adjustments (Δ*E*_ZPE_ – *T*Δ*S*), simplifying Δ*G*_H_ to Δ*E* +0.24 eV.^42,43^

Comprehensive DFT calculations were conducted using the Vienna Ab-initio Simulation Package (VASP), employing the projector-augmented wave (PAW) method with the Perdew–Burke–Ernzerhof (PBE) functional^44−46^ to obtain the most stable structures. Subsequently, Δ*E* values were obtained via PBE with the Grimme D3(BJ) dispersion correction.^47^ Fe_2_P exists in two different structures: hexagonal and orthorhombic. To model Δ*G*_H_ on hexagonal Fe_2_P (111) surfaces with Fe_24_P_12_ termination, a periodically repeated slab with 4 layers and a 15 Å vacuum was employed, using cell parameters *a* = *b* = 13.4 Å, *c* = 18.3 Å, α = β = 90°, γ = 96.6°. For orthorhombic Fe_2_P (210) surfaces with Fe_11_P_7_ termination, a periodically repeated slab with 4 layers and 15 Å vacuum was used, with cell parameters *a* = 14.3 Å, *b* = 10.1 Å, *c* = 17.5 Å, and α = β = γ = 90°. In both models, atoms in the bottom layer were fixed to their bulk positions to simulate the bulk structure accurately. A plane wave cutoff energy of 520 eV and a Γ-centered 5 × 5 × 1 grid of k-points were utilized for all calculations. In addition to studying a 3.13% composition of each dopant, the influence of Mo at different doping levels (6.26 and 9.39%) was investigated on the orthorhombic Fe_2_P (210) surface. For higher Mo concentrations, the same cell parameters were employed with two and three Fe atoms substituted by Mo atoms, respectively. An intermediate (4.69% Mo) composition was also investigated by constructing a larger unit cell (*a* = 28.6 Å, *b* = 10.1 Å, and *c* = 17.5 Å), substituting three Fe atoms with three Mo atoms and determining the most stable structure.

## Results and Discussion

### Theoretical Studies on Fe_2_P and Fe_2–*x*_Mo_*x*_P

**Figure 1:**
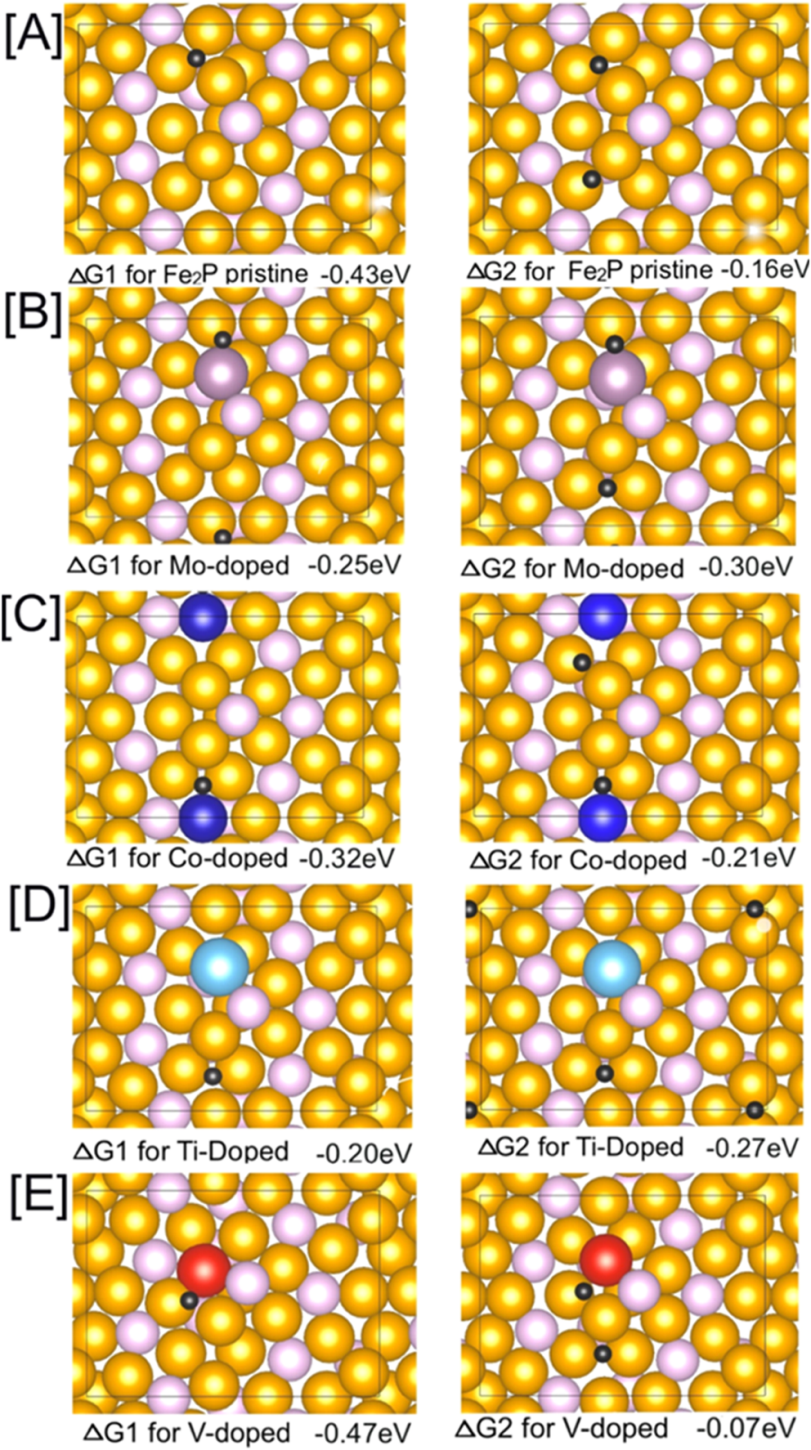
First and second Δ*G*_H_ values on (A) pristine Fe_2_P along with (B) Mo-, (C) Co-, (D) Ti-, and (E) V-doped orthorhombic Fe_2_P (210) surfaces with Fe_11_P_7_ termination. The concentration of heteroatoms was fixed at 3.13% for all structures. Fe atoms are colored yellow, P atoms pink, H atoms black, and the dopant atoms various colors.

To evaluate the HER performance of Fe_2_P and its doped variants, we conducted DFT calculations, focusing on Δ*G*_H_ at various adsorption sites on the orthorhombic Fe_2_P (210) and hexagonal Fe_2_P (111) surfaces. Δ*G*_H_ was computed at several hydrogen binding sites, including atom vertices, Fe–Fe bridges, and Fe–P bridges, to determine the most favorable hydrogen adsorption sites. The first and second Δ*G*_H_ values are key parameters in assessing the HER efficiency of pristine and heteroatom-doped Fe_2_P surfaces. Figure 1 presents the Δ*G*_H_ values and corresponding structural configurations for orthorhombic Fe_2_P and the four most promising (Mo, Co, Ti, and V) doped surfaces. The Δ*G*_H_ values of Fe_2_P doped with other heteroatoms (Cr, Cu, Mn, and Ni) and composition-dependent Δ*G*_H_ (3.13, 6.26, and 9.39%) calculations of Fe_2–*x*_Mo_*x*_P are shown in Figures S1 and S2, respectively. The Δ*G*_H_ values of pristine and transition-metal-doped (Co, Cr, Cu, Mo, Mn, Ni, V, and Ti) hexagonal Fe_2_P (111) surfaces are shown in Figure S3. The computed Δ*G*_H_ values for orthorhombic Fe_2_P (210) and hexagonal Fe_2_P (111) surfaces are summarized in Tables 1 and S1, respectively.

**Table 1:** First and Second Δ*G*_H_ Values on Pristine and Doped Orthorhombic Fe_2_P (210) Surfaces with Fe_11_P_7_ Terminations for Eight Transition Metal Dopants Studied at a Dopant Concentration of 3.13%^a^

	Δ*G*_H1_ (eV)	Δ*G*_H2_ (eV)
pristine	–0.43	–0.16
Co	–0.32	–0.21
Cr	–0.94	–0.69
Cu	–0.41	–0.94
Mn	–0.36	–0.31
Mo	–0.25	–0.30
Ni	–1.18	–0.72
V	–0.47	–0.07
Ti	–0.20	–0.27
Mo (4.69%)	–0.20	0.06
Mo (6.26%)	–0.92	0.18
Mo (9.39%)	–0.29	–0.83

a Δ*G*_H_ values of Mo-doped structures at 4.69, 6.26, and 9.39% Mo compositions are also shown.

Notably, the first and second Δ*G*_H_ values are negative for all orthorhombic Fe_2_P (210) surfaces, with the exception of the second Δ*G*_H_ on the Mo-doped surfaces at 4.69 and 6.26% compositions. Through a computational investigation of various adsorption sites, including atom vertices, Fe–Fe bridges, and Fe–P bridges, it was determined that the lowest-energy position for the first hydrogen atom adsorption on the pristine surface is at the center of a triangle formed by three Fe atoms. The most stable binding position for the second H atom is on the Fe–Fe bridge site. The overall |Δ*G*_H_| binding, represented as |Δ*G*_H1_| + |Δ*G*_H2_| is 0.59 eV in pristine Fe_2_P. When one Fe atom is replaced with Co, Mo, Ti, Cu, or Mn, the first ΔG_H_ becomes weaker (closer to zero), indicating an enhancement in the HER activity. Conversely, admixing of Ni, Cr, and V results in a stronger (more negative) first Δ*G*_H_, which is detrimental to HER. For the second Δ*G*_H_, all dopants except V increase the binding strength of the second H, while V causes a decrease, making it more favorable for HER. The overall |Δ*G*_H_| slightly decreases with the doping of Mo, V, Co, and Ti, with values of 0.55, 0.54, 0.53, and 0.47 eV, respectively. Among all dopants investigated, Ti emerges as the best dopant with an overall |Δ*G*_H_| of 0.47 eV. Ti significantly decreases the first Δ*G*_H_ (on the Fe–Ti bridge site) and only slightly increases the second Δ*G*_H_ (on the Fe–Fe bridge site), resulting in the smallest overall |Δ*G*_H_| among all dopants. Considering the theoretical calculation uncertainty of ∼0.1 eV, Mo, V, Co, and Ti all show potential as promising dopants for Fe_2_P, as illustrated in Figure 1. Because of challenges in synthesizing certain transition-metal-doped Fe_2_P nanostructures (e.g., Ti-doped Fe_2_P) with control over structure, morphology, and composition, the experimental efforts were focused on the synthesis and HER activity investigation of the Fe_2–*x*_Mo_*x*_P system.

As the Mo concentration increases from 3.13 to 9.39%, the surface properties undergo significant changes. The total |Δ*G*_H_| decreases from 0.55 eV at 3.13% to the lowest value of 0.26 eV at 4.69%, marking the optimal performance among all investigated surfaces. However, as the Mo concentration exceeds 4.69%, the total |Δ*G*_H_| increases rapidly. This change is attributed to the destruction of the Mo–Fe bridge, which alters the adsorption sites for both the first and second hydrogen atoms. Specifically, the first hydrogen adsorption shifts from the Mo–Fe bridge to the Fe triangle, while the second hydrogen shifts from the Fe–Fe bridge to the Fe triangle. These shifts result in a substantial increase in the first Δ*G*_H_ binding to −0.92 eV and a slightly repulsive second Δ*G*_H_ binding of 0.18 eV. Consequently, the overall |Δ*G*_H_| doubles, rising from 0.55 eV at 3.13% Mo to 1.10 eV at 6.26% Mo. At 9.39% Mo, further distortions and the destruction of Fe triangles decrease the first hydrogen binding and increase the second hydrogen binding, leading to an overall |Δ*G*_H_| of 1.12 eV and further degradation of the catalytic efficiency. These results align with experimental observations, confirming that HER activity peaks at approximately 4.69% Mo concentration.

For the phase-pure hexagonal Fe_2_P (111) surface, the overall |Δ*G*_H_| binding is 0.92 eV, significantly higher than the 0.59 eV observed on the orthorhombic Fe_2_P (210) surface, indicating a higher HER performance on the orthorhombic Fe_2_P (210) surface. Additionally, dopant atoms have a minimal effect on the adsorption activity of the hexagonal Fe_2_P (111) surface, with variations in free energy limited to ∼0.03 eV. Computational studies predict that the most favorable absorption sites for the first and second hydrogen atoms on hexagonal Fe_2_P (111) are located at the centers of Fe triangles, which are distant from the dopant atoms. This spatial separation results in a negligible influence of dopants on hydrogen absorption. This observation can be attributed to the differences in surface electronic structures between the hexagonal Fe_2_P (111) and orthorhombic Fe_2_P (210) surfaces. On the hexagonal Fe_2_P (111) surface, dopants do not occupy the Fe triangle sites, whereas on the orthorhombic Fe_2_P (210) surface, dopants tend to occupy and disrupt these sites, making it easier to modify the surface electronic structure of the resulting surface. As dopant concentration increases on hexagonal surfaces, more Fe atoms are replaced by dopants, eventually occupying the Fe triangle sites. However, this also induces severe surface fluctuations, leading to structural collapse in computational models. As a result, increasing dopant concentration is not a viable strategy for enhancing HER performance on the hexagonal Fe_2_P surface and instead leads to a structural transition from the hexagonal to the orthorhombic phase, consistent with experimental observations. These findings offer valuable insights into the role of surface structure and dopant placement in catalytic efficiency, providing guidance for future research on effective dopants for Fe_2_P.

### Synthesis and Characterization of Fe_2_P and Fe_2–*x*_Mo_*x*_P NRs

**Figure 2:**
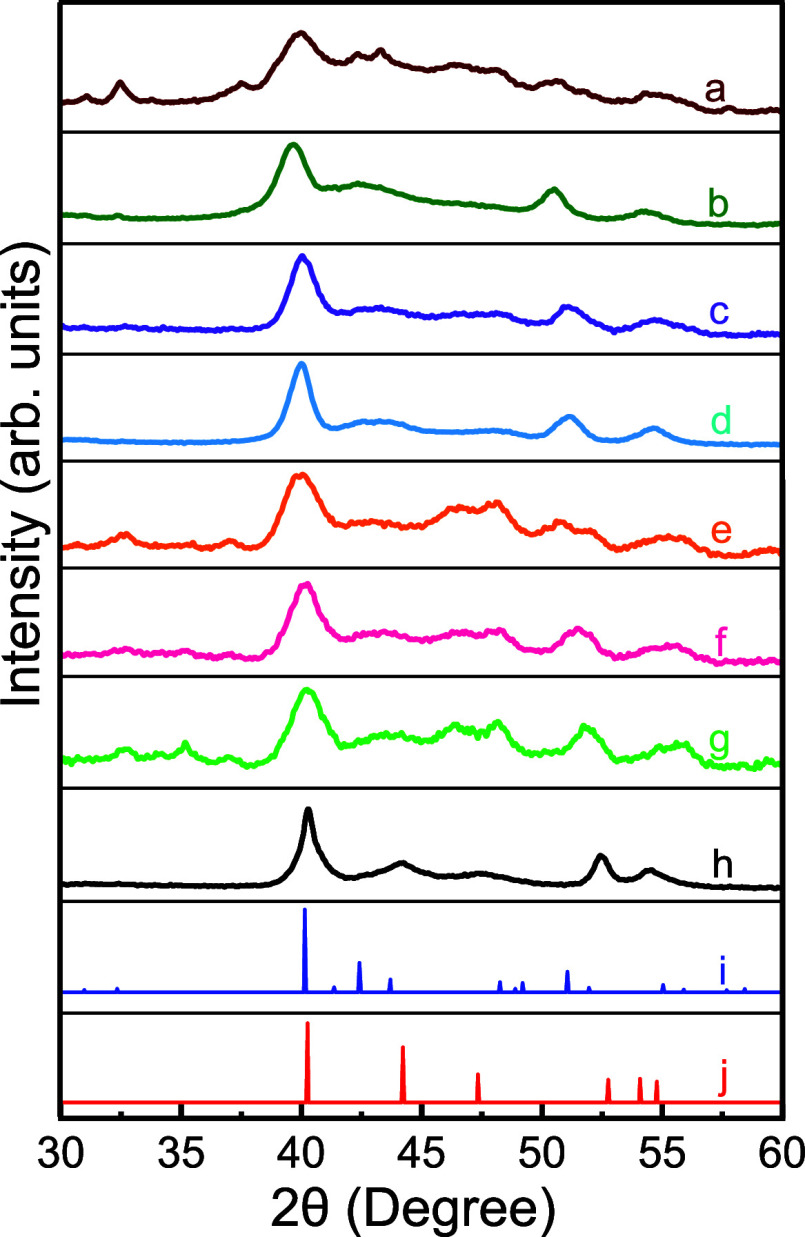
PXRD patterns of (a) Fe_1.70_Mo_0.30_P, (b) Fe_1.76_Mo_0.24_P, (c) Fe_1.86_Mo_0.14_P, (d) Fe_1.89_Mo_0.11_P, (e) Fe_1.91_Mo_0.09_P, (f) Fe_1.94_Mo_0.06_P, (g) Fe_1.97_Mo_0.03_P, (h) Fe_2_P NRs along with (i) orthorhombic Fe_2_P (PDF 01-090-8789) and (j) hexagonal Fe_2_P (PDF 01-078-6747) reference patterns.

**Scheme 1:**
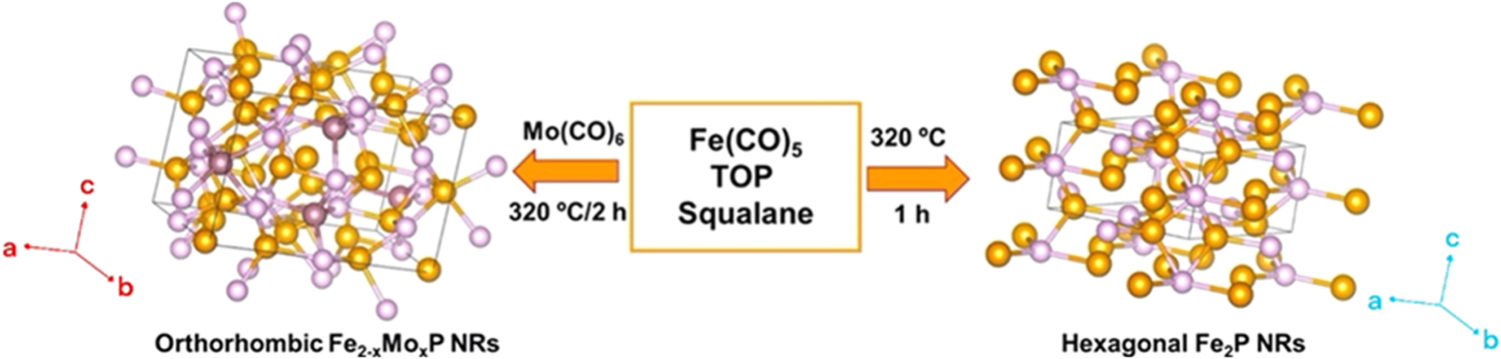
Schematic Illustration of the Synthesis of Hexagonal Fe_2_P and Orthorhombic Fe_2–*x*_Mo_*x*_P NRs

On the basis of computational predictions, hexagonal Fe_2_P (Figure S4), hexagonal and orthorhombic Fe_2–*x*_Mo_*x*_P NRs (Figure 2) with variable compositions were synthesized for a systematic HER activity investigation as a function of crystal structure and composition. The colloidal synthesis of phase-pure hexagonal Fe_2_P NRs can be achieved through thermal decomposition of Fe(CO)_5_ and TOP in alkylamine (OLA) solvents.^48^ Literature reports indicate that the nature of the iron precursor is critical for the synthesis of Fe_2_P NRs. The high reactivity of Fe(CO)_5_ allows rapid decomposition at elevated temperatures, allowing for the formation of phase-pure NRs without any undesired (Fe, FeP, or FeO_*x*_) impurities.^35^ In our experiments, a molar ratio of Fe(CO)_5_/TOP 0.46:1 was used to produce hexagonal Fe_2_P and orthorhombic and mixed-phase Fe_2–*x*_Mo_*x*_P NRs at 320 °C with a growth time of 1 and 2 h, respectively. Deviating from this optimal temperature required longer growth times at low temperatures or resulted in phase impurities at high temperatures. When Mo-doped NRs were synthesized, the addition of Mo(CO)_6_ destabilized the reaction, with the amine group of OLA forming a side product with molybdenum. Thus, squalane was used as both the solvent and surfactant to reach optimal temperatures (320 °C) and growth time (2 h) to produce Fe_2–*x*_Mo_*x*_P with NR morphology (Scheme 1).

Fe_2–*x*_Mo_*x*_P NRs were synthesized by admixing Mo(CO)_6_ and Fe(CO)_5_ with reactive phosphorus species produced by the decomposition of TOP in squalane. Additionally, the growth temperature was maintained at 320 °C, which is sufficient to produce Fe_2–*x*_Mo_*x*_P NRs. Given that Mo(CO)_6_ has melting and boiling points of 150 and 156 °C, respectively, 320 °C was sufficient to produce reactive Mo species. Structure, crystallinity, and phase purity of NRs were investigated with PXRD. The diffraction patterns of Fe_2_P and Fe_2–*x*_Mo_*x*_P NRs are shown in Figure 2, demonstrating the high crystallinity of the samples. Binary Fe_2_P NRs display a hexagonal structure, and corresponding Bragg reflections can be indexed to Barringerite (PDF 01-078-6747) reference pattern. In contrast, Fe_2–*x*_Mo_*x*_P NRs show obvious changes in structure upon the admixing of Mo. Interestingly, when the composition of Mo is increased above 3.09%, a couple of trends emerged. The first is that the diffraction patterns began to shift toward lower 2θ angles relative to binary Fe_2_P. Initially, the highest intensity (111) peak was centered at 40.2° for hexagonal Fe_2_P. With an increase in Mo composition to 14.08%, a systematic shift in (111) peak to 39.6° was observed, suggesting an expansion of the lattice, as shown in Figure S5. This shift is expected given that the atomic size of Mo (190 pm) is larger than that of Fe (156 pm).

The second trend to emerge from the PXRD data was the change in crystal structure upon admixing of Mo into hexagonal Fe_2_P. Fe_2_P has been previously shown to exhibit two distinct structure types, hexagonal and orthorhombic, with hexagonal being the most thermodynamically stable structure.^49,50^ Both hexagonal and orthorhombic Fe_2_P bulk materials can be synthesized under extreme conditions via a tin-flux reaction pathway.^49^ However, to the best of our knowledge, orthorhombic Fe_2_P has not been reported at the nanoscale. We found that upon incorporation of Mo into hexagonal Fe_2_P NRs at sufficiently high levels, the crystal structure transitions to orthorhombic Fe_2_P (PDF 01-090-8789, Allabogdanite) phase. Specifically, at 3.09% Mo composition (*x* = 0.06), as-synthesized NRs exhibit diffraction peaks that can be indexed to both hexagonal and orthorhombic Fe_2_P. The asymmetry of the main peak at 2θ = 40.2° for *x* = 0.03–0.09 compositions can be attributed to the coexistence of hexagonal and orthorhombic phases (Figure S6). When the Mo content was further increased to 5.41% (*x* = 0.11), phase-pure Fe_2–*x*_Mo_*x*_P NRs with an orthorhombic structure were obtained (Figure 2f). The expansion of the crystal lattice, owing to the size difference between Fe and Mo, likely caused the orthorhombic structure to become more favorable and stable. As the Mo content was increased further, the orthorhombic structure persisted for Fe_2–*x*_Mo_*x*_P NRs with *x* = 0.28 (14.08%). Additionally, there is no evidence of impurity phases such as hexagonal Fe_2_P, orthorhombic FeP, FeO, or MoP, suggesting that bimetallic NRs with 0.11 ≥ *x* ≤ 0.28 are phase pure. For *x* < 0.11 compositions, the presence of both hexagonal and orthorhombic Fe_2_P was observed, as shown in Figure S7.

Due to the observed phase transition, both crystal structures were extensively studied via DFT to evaluate their HER performance. The results revealed that hexagonal Fe_2_P not only exhibits suboptimal Δ*G*_H_ values for hydrogen adsorption but also demonstrates a limited response to heteroatom doping. Doping with synergistic transition metals, which typically enhances HER activity, had a minimal effect on improving the overall |Δ*G*_H_| of hexagonal Fe_2_P. This is likely due to the rigid electronic structure and atomic arrangement of the hexagonal phase, which restricts dopant-induced modifications of the HER active sites. In contrast, orthorhombic Fe_2_P showed significantly improved adaptability to heteroatoms, with dopants notably improving the surface electronic structure and the overall |Δ*G*_H_|. DFT calculations indicated that Mo doping introduced favorable changes in Δ*G*_H_, creating active sites that were more conducive to hydrogen adsorption and desorption, thereby improving the overall catalytic efficiency of orthorhombic Fe_2_P. These findings suggest that orthorhombic Fe_2_P is inherently more flexible and responsive to dopant-induced modifications, making it a superior candidate for HER applications. Elemental composition of as-synthesized Fe_2_P and Fe_2–*x*_Mo_*x*_P NRs was investigated with SEM-EDS, and the results are shown in Table 2. The average Mo composition varies from *x* = 0.03–0.28, corresponding to 1.72–14.08% Mo. The experimental Mo composition depends on the nominal molar ratio of the Fe and Mo precursors, although a systematic correlation was not observed. For instance, 5–6% nominal Mo composition produced NRs with experimental Mo content 6.09–11.95% range and 1–4% nominal concentration produced NRs with Mo content 1.72–14.08% range. While this trend was held for most samples, there were a few outliers where the nominal concentration varied notably from the experimental compositions (Table 2).

**Table 2:** Nominal and Experimental Compositions and Average Lengths and Widths of Fe_2–*x*_Mo_*x*_P NRs Produced via Colloidal Synthesis

		elemental composition (SEM-EDS)^a^				
sample name	nominal composition (Mo)	Fe %	Mo %	P %	NR length, nm (TEM)^b^	NR width, nm (TEM)^b^
Fe_2_P	0%	69.16	0.00	30.84	74.2 ± 21.1	4.2 ± 1.1
Fe_1.97_Mo_0.03_P	1.0%	67.63	1.72	30.65	59.5 ± 39.3	3.8 ± 1.0
Fe_1.94_Mo_0.06_P	8.0%	62.21	3.09	34.70	85.2 ± 43.9	4.0 ± 1.3
Fe_1.91_Mo_0.09_P	0.8%	61.16	4.58	34.26	51.2 ± 23.2	4.9 ± 1.0
Fe_1.88_Mo_0.12_P	6.0%	51.12	6.09	42.79	50.9 ± 22.1	5.5 ± 1.1
Fe_1.88_Mo_0.12_P	3.0%	53.24	6.20	40.56	57.6 ± 16.8	4.2 ± 1.5
Fe_1.86_Mo_0.14_P	5.0%	51.59	7.05	41.36	65.1 ± 32.8	4.6 ± 1.7
Fe_1.77_Mo_0.23_P	4.0%	50.32	11.80	37.73	92.4 ± 43.8	6.3 ± 1.8
Fe_1.75_Mo_0.25_P	8.0%	49.30	12.60	38.55	75.9 ± 49.5	5.7 ± 1.5
Fe_1.72_Mo_0.28_P	3.0%	39.31	14.08	46.61	63.1 ± 26.9	4.7 ± 0.9

a Elemental compositions were obtained by SEM-EDS. Each composition was determined by averaging the atomic percent of elements obtained from five individual spots per sample.

b Average length and width of NRs were calculated from ca. 150–200 individual NRs obtained from multiple LRTEM and HRTEM images.

**Figure 3:**
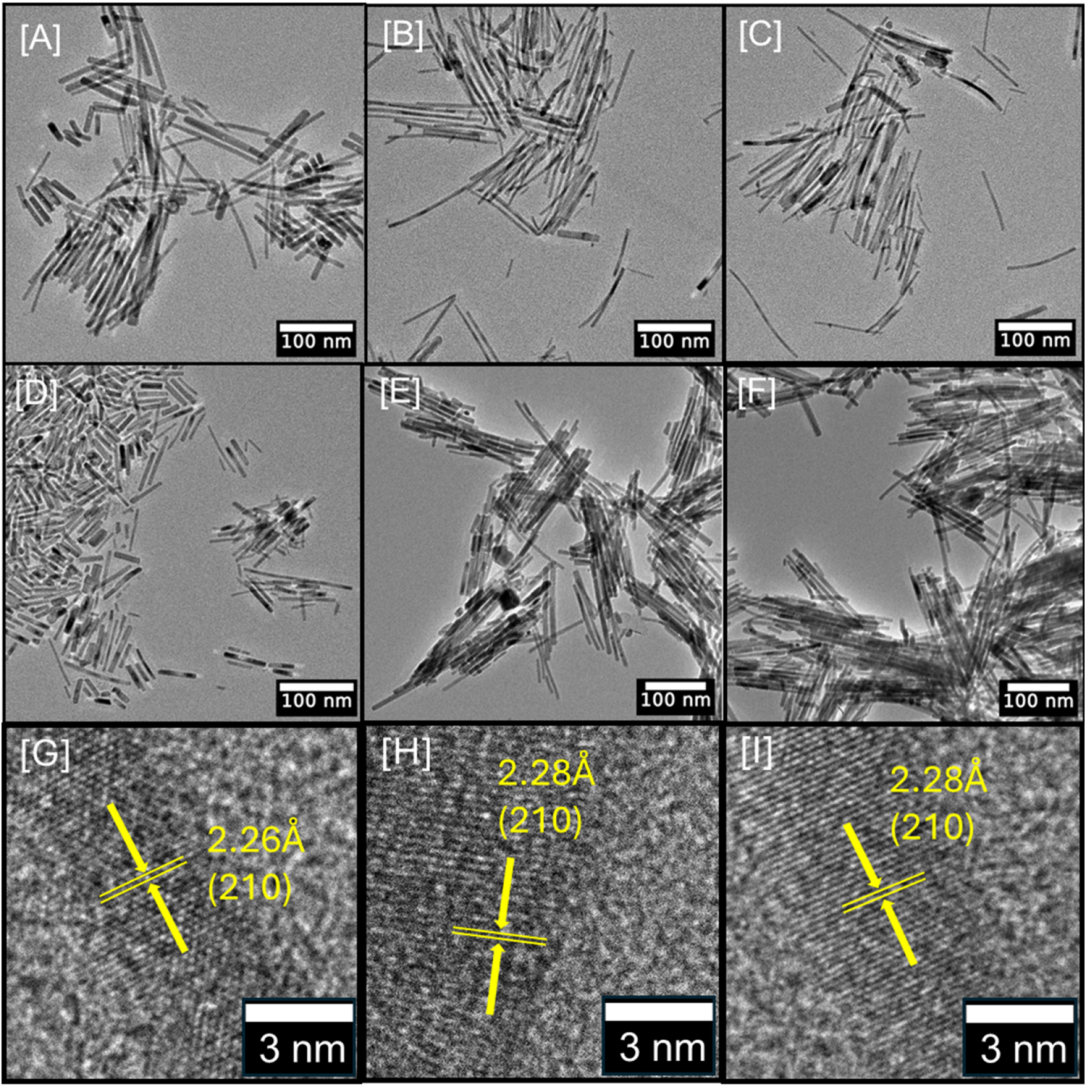
Low-resolution TEM images of (A) hexagonal Fe_2_P NRs along with (B) Fe_1.97_Mo_0.03_P, (C) Fe_1.94_Mo_0.06_P, (D) Fe_1.86_Mo_0.14_P, (E) Fe_1.76_Mo_0.24_P, and (F) Fe_1.75_Mo_0.25_P NRs. HRTEM images of (G) Fe_1.88_Mo_0.12_P, (H) Fe_1.77_Mo_0.23_P, and (I) Fe_1.75_Mo_0.25_P.

**Figure 4:**
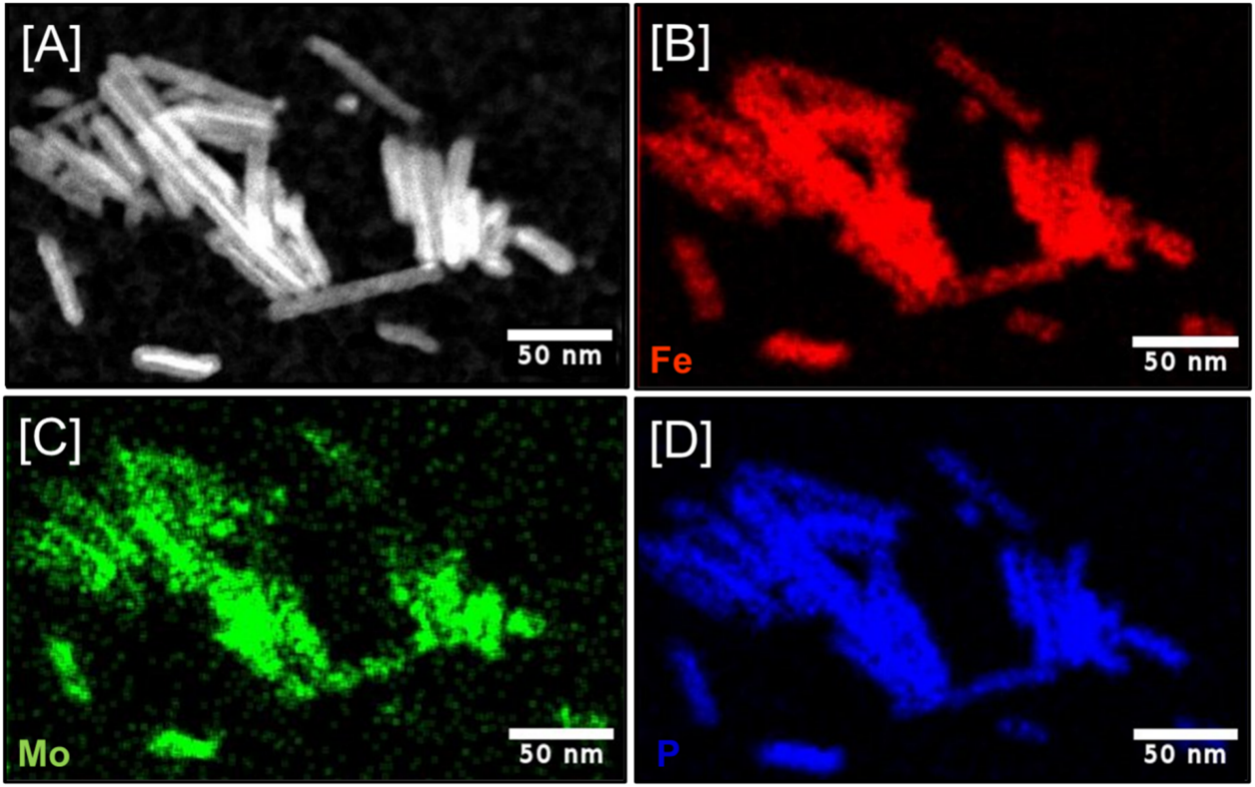
(A) STEM image and STEM-EDS elemental maps of (B) Fe, (C) Mo, and (D) P recorded from Fe_1.88_Mo_0.12_P NRs displaying the structural homogeneity of the particles.

Fe_2_P nanostructures have been shown to adopt anisotropic morphologies (rods or wires) as opposed to spherical particles.^35^ This set Fe_2_P apart from other iron phosphides that tend to form spheres and therefore have to be grown into anisotropic particles synthetically.^26,27,29^ The size and shape of Fe_2–*x*_Mo_*x*_P NRs were investigated with TEM, and the corresponding images are shown in Figure 3. Binary Fe_2_P exhibited solid NR morphology with an average length and width of 74.2 ± 21.1 and 4.2 ± 1.1 nm, respectively. These NRs have been reported to display a [001] growth direction along the (002) plane,^35,51^ which is consistent with the increased intensity of the (002) peak observed in the corresponding PXRD pattern (Figure S4). In contrast, for Fe_2–*x*_Mo_*x*_P NRs, length varies widely across all samples, with the average ranging from 85.2 ± 43.9 to 63.1 ± 26.9 nm for *x* = 0.06 (3.09%) to 0.28 (14.08%) compositions, respectively. The width of Fe_2–*x*_Mo_*x*_P NRs remains more consistent, with an average ranging from 3.8 ± 1.0 to 6.3 ± 1.8 nm for *x* = 0.03 (1.72%) to 0.23 (11.8%) compositions, respectively. These values for Fe_2_P NRs fall within the same length and width ranges as for Fe_2–*x*_Mo_*x*_P NRs. This indicates that the incorporation of Mo has no effect on the morphology and size of the NRs. It is worth noting that previous reports on colloidally synthesized Ni_1–*y*_Mo_*y*_P NCs showed Mo segregation above 6.0% Mo composition, leading to the formation of Janus-type particles consisting of Ni–P- and Mo–P-rich regions.^52^ However, with colloidal Fe_2–*x*_Mo_*x*_P NRs, all samples produced were homogeneous and maintained the original morphology of hexagonal Fe_2_P NRs despite a dramatic change in the crystal structure. The elemental maps of Fe_1.88_Mo_0.12_P NRs were recorded using STEM-HADDF and are shown in Figure 4B–D. These maps confirm the presence of Fe, Mo, and P in all samples and the homogenous distribution of elemental constituents throughout the NRs with no evidence of segregation. This has been confirmed with Fe_2–*x*_Mo_*x*_P NRs with variable compositions up to *x* = 0.25 (12.60%) as shown in Figures S8 and S9. The lattice fringes calculated from HRTEM images of Fe_1.88_Mo_0.12_P, Fe_1.77_Mo_0.23_P, and Fe_1.75_Mo_0.25_P NRs demonstrate values from 2.26 to 2.28 Å that can be indexed to the (210) plane of orthorhombic Fe_2_P. These *d* spacings cannot be correlated to hexagonal Fe_2_P, suggesting that the hexagonal to orthorhombic transition had occurred prior to *x* = 0.12, consistent with PXRD studies (Figure 2).

**Figure 5:**
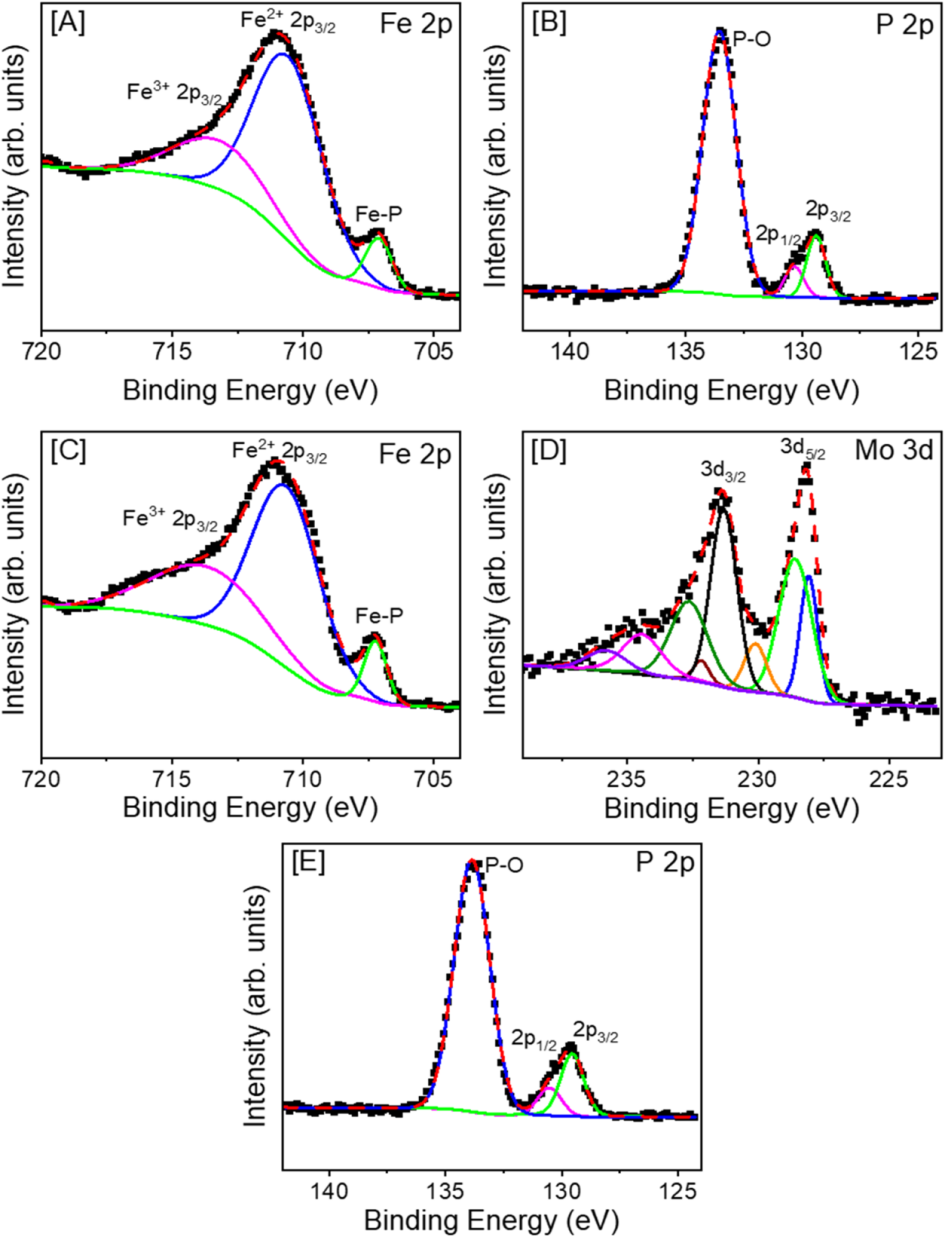
XPS spectra of the (A) Fe 2p and (B) P 2p regions of hexagonal Fe_2_P and (C) Fe 2p, (D) Mo 3d, and (E) P 2p regions of Fe_1.81_Mo_0.19_P NRs. Black solid and dashed lines represent experimental data, and colored lines are fitted deconvolutions. Samples were annealed for 2 h at 450 °C under a 5% H_2_:Ar atmosphere.

XPS spectra were recorded for Fe_2_P and Fe_2–*x*_Mo_*x*_P NRs to determine the oxidation states of surface species (Figure S10). For both Fe_2–*x*_Mo_*x*_P and Fe_2_P NRs, small positive shifts in the Fe 2p and Mo 3d binding energies are expected, in addition to negative shifts in P 2p binding energies. These shifts indicate changes in oxidation states, which are likely to originate from differences in the electronegativity of metal (Fe and Mo) and nonmetal (P) elements.^53−55^ In the Fe 2p region of hexagonal Fe_2_P (Figure 5A), the deconvoluted peak at 707.19 eV, which is shifted slightly to higher energy than expected (706.8 eV), can be attributed to the Fe–P bond consistent with the literature.^34,56^ Additionally, the deconvoluted peaks at 710.59 and 713.26 eV can be attributed to oxidized iron species (Fe^2+^ and Fe^3+^), respectively, presumably originating from surface oxidation upon exposure to ambient atmosphere prior to examination.^54^ The P 2p region of hexagonal Fe_2_P (Figure 5B) showed a deconvoluted doublet at 129.49 and 130.28 eV, corresponding to metal phosphides (P^δ−^)^55^ and a peak at 133.56 eV corresponding to surface phosphate species typically observed in transition metal phosphides.^52^

Bimetallic Fe_1.91_Mo_0.09_P (Figure S11) and Fe_1.81_Mo_0.19_P (Figure 5) NRs displayed similar characteristics in the Fe 2p and P 2p regions. For Fe_1.81_Mo_0.19_P NRs, the deconvoluted peak at 707.07 eV corresponds to the iron phosphide bond (Fe–P), similar to Fe_2_P. The deconvoluted peaks at 710.77 and 713.95 eV indicate the presence of oxidized surface species (Fe^2+^ and Fe^3+^) likely originating from sample handling in the ambient atmosphere. The P 2p region exhibits a deconvoluted doublet at 129.57 and 130.58 eV that can be attributed to metal phosphide bonds (P^δ−^), along with a deconvoluted singlet at 133.87 eV, which corresponds to surface phosphate species.^55^ In the Mo 3d spectrum, the deconvoluted 3d_5/2_ and 3d_3/2_ spectra display four doublets with binding energies of 228.12 and 231.32 eV, which are slightly higher than metallic Mo^0^ (227.8 and 231.0 eV), indicating the presence of partially charged Mo^δ+^ species.^57,58^ The remaining three doublets at 228.63 and 232.22 eV, 230.14 and 234.5 eV, and 232.63 and 235.85 eV can be attributed to Mo^4+^ (MoO_2_),^48,54^ Mo^5+^ (Mo_*x*_O_*y*_), and Mo^6+^ (MoO_3_), respectively, consistent with the literature.^57,58^ These oxide species are likely to originate from surface oxidation upon handling of samples in an ambient atmosphere. Several conclusions can be formulated from the analysis of the binding energies. First, the binding energies of Fe^δ+^ species decreased from 707.19 to 707.07 eV for hexagonal Fe_2_P and orthorhombic Fe_1.81_Mo_0.19_P, respectively, indicating that Fe^δ+^ became less positive as the Mo concentration increased to *x* = 0.19. Conversely, the binding energies of P^δ−^ species increased from 129.49 and 130.28 eV to 129.57 and 130.58 eV for hexagonal Fe_2_P and orthorhombic Fe_1.81_Mo_0.19_P, respectively, suggesting that the partial negative charge of P^δ−^ decreases with Mo incorporation. These shifts observed with Fe and P surface moieties indicate that Mo modifies the surface chemistry of bimetallic NRs. These findings are consistent with computational calculations, which suggest substantial changes in the Δ*G*_H_ values, with the potential to improve the HER performance.

### Electrocatalytic Activity of Fe_2_P and Fe_2–*x*_Mo_*x*_P NCs for HER

The HER activity and stability of Fe_2_P and Fe_2–*x*_Mo_*x*_P NRs were evaluated with LSV and chronopotentiometry and compared with commercial Pt/C (10 wt %) catalyst. For the working electrode fabrication, catalyst inks were produced by sonicating a mixture of catalyst powder (Fe_2–*x*_Mo_*x*_P NRs or Pt/C), isopropyl alcohol, ultrapure water, and Nafion. Nafion was selected as a binding agent because it acts as both a binder to hold catalyst NRs to the electrode surface and a proton conductor. Thus, this facilitates efficient ion transport within the electrode and enhances overall electrochemical performance and stability. Ti foil was used as the substrate for electrode fabrication due to its high mechanical strength and excellent conductivity. Surface-bound organic ligands (OLA, TOP, and squalane) hinder catalytic activity by decreasing the number of available active sites. Thus, working electrodes were annealed at 450 °C for 2 h in 5% H_2_/Ar to remove residual ligands, enhance ohmic conductivity, and improve catalyst adhesion to the Ti substrate. This annealing procedure did not alter the crystal structure (Figure S12) or the morphology of the NRs, although significant agglomeration was observed owing to the loss of passivating surface ligands (Figure S5).

**Figure 6:**
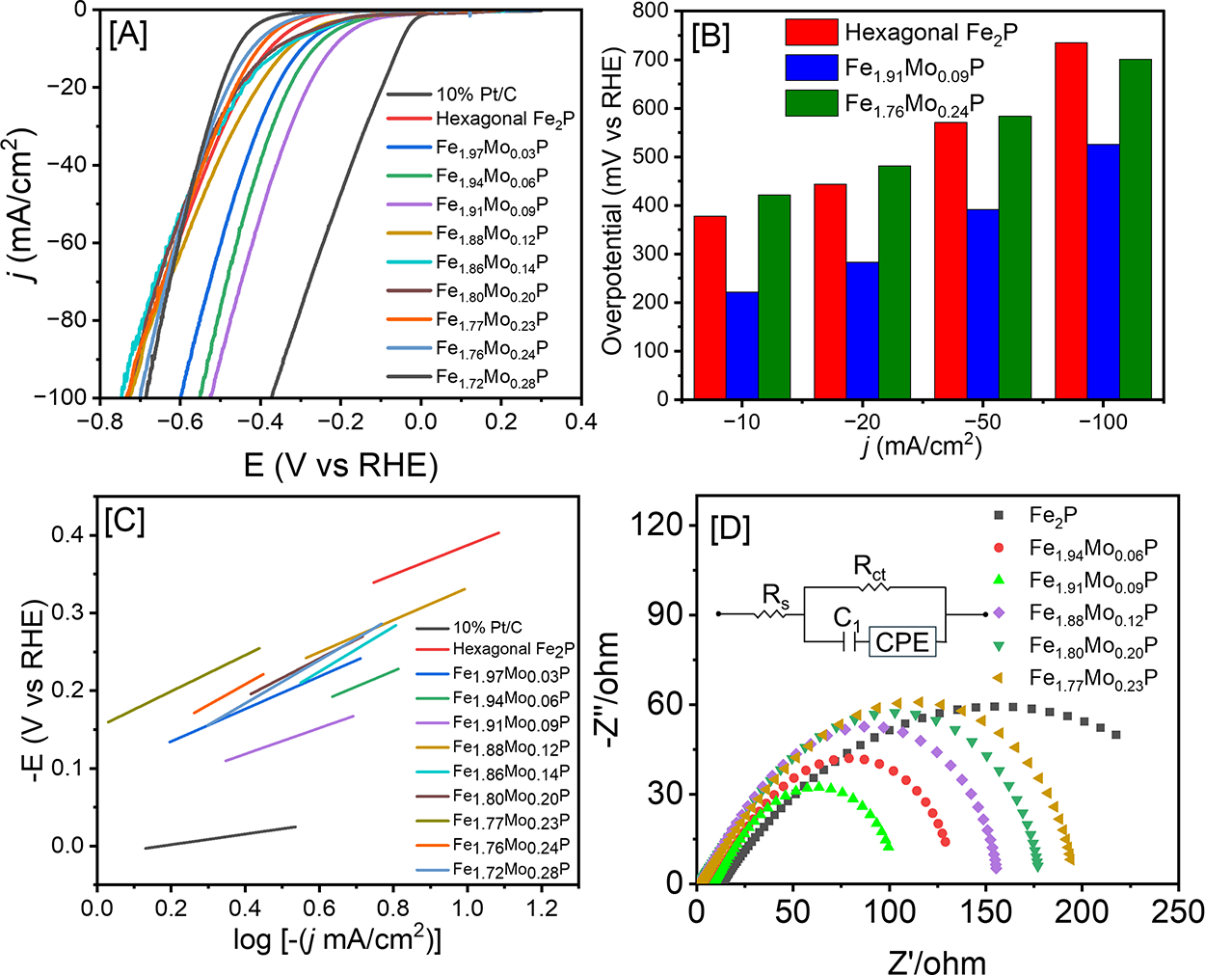
(A) Polarization plots of hexagonal Fe_2_P NRs, hexagonal and orthorhombic Fe_2–*x*_Mo_*x*_P NRs, and commercial Pt/C catalysts in 1 M KOH at a 5 mV/s scan rate. (B) Comparison of overpotentials of Fe_1.91_Mo_0.09_P and Fe_1.76_Mo_0.24_P NRs and hexagonal Fe_2_P at *j* = −10, −20, −50, and −100 mA/cm^2^. (C) Tafel plots of Fe_2–*x*_Mo_*x*_P NRs and commercial Pt/C derived from LSV in 1 M KOH. (D) Nyquist plots of binary Fe_2_P, Fe_1.94_Mo_0.06_P, Fe_1.91_Mo_0.09_P, Fe_1.88_Mo_0.12_P, Fe_1.80_Mo_0.20_P, and Fe_1.77_Mo_0.23_P with an equivalent circuit used to simulate the Nyquist plots.

The effects of Mo doping on the HER activity and stability of Fe_2_P NRs were investigated in alkaline (1 M KOH) electrolytes using LSV and EIS. Literature studies report high HER activity of heteroatom-doped (Ni) Fe_2_P nanostructures^32^ and thin films^29^ in acid electrolytes (Table S2). However, a handful of studies reported the HER activity of Fe_2_P in alkaline electrolytes.^59,60^ Therefore, a basic electrolyte medium was chosen for the HER activity and stability experiments. As shown in Figure 6A, binary Fe_2_P NRs require a higher overpotential (η_–10 *=*_ 378 mV) to reach *j* = −10 mA/cm^2^ compared to benchmark Pt/C (η_–10_ = 61 mV), NiMo alloy (η_–10_ = 62 mV),^58^ and Ni/C (η_–10_ = 94 mV)^61^ catalysts, suggesting a lower HER activity. However, the homogeneous admixture of Mo instigated a notable increase in HER activity, as shown in Figure 6B. With increasing concentration of Mo from *x* = 0 to 0.09, a prominent decrease in η_–10_ from 378 to 222 mV was observed, suggesting an increase in HER activity (Figure 6A–C). This decrease in overpotentials can be correlated to the theoretical findings, which suggest that orthorhombic Fe_2_P is more catalytically active than hexagonal Fe_2_P. Therefore, the structural transformation upon Mo doping caused the shift toward the more favorable orthorhombic Fe_2_P and higher HER performance. However, with further increasing the Mo concentration (*x* = 0.12–0.28), a notable increase in η_–10_ occurred, and consequently, the highest Mo-containing Fe_1.72_Mo_0.28_P NRs displayed the lowest HER activity (η_–10_ = 459 mV) among all samples investigated (Table 3). This observation can be attributed to a decrease in active sites with increasing Mo composition above a critical concentration (*x* = 0.09). Hence, composition-dependent Δ*G*_H_ calculations were conducted to determine the influence of dopants (Mo) on the HER activity. The orthorhombic Fe_1.94_Mo_0.06_P (3.13% Mo) showed Δ*G*_H_ values of −0.25 and −0.30 eV for the Δ*G*_H1_ and Δ*G*_H2_ hydrogen adsorption steps. Notably, Fe_1.91_Mo_0.09_P (4.69% Mo) exhibited the lowest Δ*G*_H_ values, at −0.20 and 0.06 eV for each step, indicating the highest catalytic activity among all samples investigated. In comparison, Fe_1.88_Mo_0.12_P (6.26% Mo) showed Δ*G*_H_ values of −0.92 and 0.18 eV for the first and second hydrogen adsorption steps, while Fe_1.86_Mo_0.14_P (9.39% Mo) demonstrated Δ*G*_H_ values of −0.29 and −0.83 eV for the two steps. These results underscore a systematic decrease in HER activity as Mo concentration increases, with orthorhombic Fe_1.91_Mo_0.09_P showing optimal HER performance (Figure S2). At different current densities, *j* = −10, −20, −50, and −100 mA/cm^2^, the trends observed in the LSV data indicated that the highest performing Fe_1.91_Mo_0.09_P NRs displayed the lowest η values at all *j* values (Figure 6B and Table 3). At higher *j* values, the η_–50_ of Fe_2–*x*_Mo_*x*_P NRs with *x* = 0.12–0.28 showed much less variation, and η_–100_ decreased to 687 mV for Fe_1.72_Mo_0.28_P composition, while other samples (*x* = 0.12–0.24) show η_–100_ > 700 mV.

**Table 3:** Comparison of Elemental Compositions, HER Overpotentials at Different Current Densities, Tafel Slopes, and Charge Transfer Resistance of Fe_2_P and Fe_2–*x*_Mo_*x*_P NR Catalysts^a^

	overpotential (η) at different current densities (mA/cm^2^)^b^					
sample name	η_–10_	η_–20_	η_–50_	η_–100_	Tafel slope (mV/dec)	charge transfer resistance, *R*_ct_ (Ω)
Fe_2_P	378	444	570	735	189.45 ± 0.57	202.2
Fe_1.97_Mo_0.03_P	298	361	470	599	209.21 ± 0.47	
Fe_1.94_Mo_0.06_P	267	329	433	553	199.81 ± 0.39	110.8
Fe_1.91_Mo_0.09_P	222	282	391	526	167.08 ± 0.23	91.1
Fe_1.88_Mo_0.12_P	331	407	551	726	206.65 ± 0.26	153.5
Fe_1.86_Mo_0.14_P	346	444	586	747	231.00 ± 2.40	
Fe_1.80_Mo_0.20_P	363	455	589	734	246.23 ± 1.60	175.2
Fe_1.77_Mo_0.23_P	376	462	579	734	233.08 ± 0.14	191.4
Fe_1.76_Mo_0.24_P	422	481	584	701	267.40 ± 0.11	
Fe_1.72_Mo_0.28_P	459	500	581	687	278.33 ± 0.17	
Pt/C	58	97	209	373	68.53 ± 0.51	

a Elemental composition of Fe, Mo, and P was obtained by SEM-EDS. Each composition was determined by averaging the weight percent of elements obtained from five individual spots per sample.

b Overpotential values were calculated from LSV-derived polarization plots.

The rate kinetics of the HER can follow two possible reaction mechanisms under alkaline conditions: the Volmer–Tafel mechanism or the Volmer–Heyrovsky mechanism. The initial step of the reaction, the Volmer step, is the one-electron reduction of a water molecule and the adsorption of H* on the catalyst surface (Volmer reaction, H_2_O + e^–^ → H* + OH^–^). This step is followed by either the Heyrovsky step (H_2_O + H* + e^–^ → H_2_ + OH^–^) or the Tafel step (2H* → H_2_) to produce H_2_^62,63^ Hence, Tafel slope calculations can be used to gain insight into HER kinetics (Figure 6C). Under alkaline conditions, when the Tafel slope reaches or exceeds 120 mV/dec, the rate-determining step is considered to be the Volmer adsorption step. In contrast, when the Tafel slope reaches 40 mV/dec, the Heyrovsky step is considered the rate-determining step.^64−66^ Tafel slopes were estimated from polarization curves and yielded values from 189.45 ± 0.57, 199.81 ± 0.39, and 167.08 ± 0.23 mV/dec for Fe_2_P, Fe_1.94_Mo_0.06_P, and Fe_1.91_Mo_0.09_P NRs, respectively. These values are higher than those obtained for commercial Pt/C (68.53 ± 0.51), NiMo alloy (61.4 mV/dec),^58^ and Ni/C (52 mV/dec)^61^ standards, suggesting slower HER kinetics for Fe_2–*x*_Mo_*x*_P NRs. The higher Tafel slopes observed for Fe_2–*x*_Mo_*x*_P NRs can also be attributed to deviations from the ideal Tafel behavior. Nevertheless, the computed slopes can be assigned to a Volmer–Heyrovsky HER mechanism, with the Volmer electrosorption being the rate-determining step. Similar slopes were obtained for mixed-phase Fe_1.97_Mo_0.03_P and Fe_1.94_Mo_0.06_P NRs, where a hexagonal to orthorhombic transition was noted, indicating slower HER kinetics in comparison to Fe_2_P. However, a volcano trend in slope values was obtained with the lowest slope for the highest performing Fe_1.91_Mo_0.09_P NRs, consistent with composition-dependent Δ*G*_H_ studies and an overall improvement in HER kinetics for the optimally doped sample (4.58%). With a further increase in Mo composition, the Tafel slopes increased concurrently, leading to a sluggish HER activity.

Additionally, EIS was used to investigate the variation in charge transfer resistance as a function of the NR composition. Fe_1.94_Mo_0.06_P, Fe_1.91_Mo_0.09_P, Fe_1.88_Mo_0.12_P, Fe_1.80_Mo_0.20_P, and Fe_1.77_Mo_0.23_P NRs were studied along with Fe_2_P NRs to investigate the influence of dopants on electrochemical impedance. Using an equivalent circuit, shown in the inset of Figure 6D, the Nyquist plots were fitted to extract the charge transfer resistance (*R*_ct_) values. The Fe_2_P, Fe_1.94_Mo_0.06_P, Fe_1.91_Mo_0.09_P, Fe_1.88_Mo_0.12_P, Fe_1.80_Mo_0.20_P, and Fe_1.77_Mo_0.23_P NRs showed *R*_ct_ values of 202.2, 110.8, 91.1, 153.5, 175.2, and 191.4 Ω, respectively. In general, higher *R*_ct_ values correspond to slower charge transfer. Therefore, the decrease in *R*_ct_ with increasing Mo from *x* = 0 to 0.09 suggests a faster faradaic response and more favorable HER kinetics for the highest performing Fe_1.91_Mo_0.09_P NRs. With further increasing Mo composition (*x* > 0.09), the *R*_ct_ increases again, suggesting a slower faradaic response and unfavorable HER kinetics, which is consistent with the increase η_–10_ observed for NRs above optimal Mo composition (4.58%).

**Figure 7:**
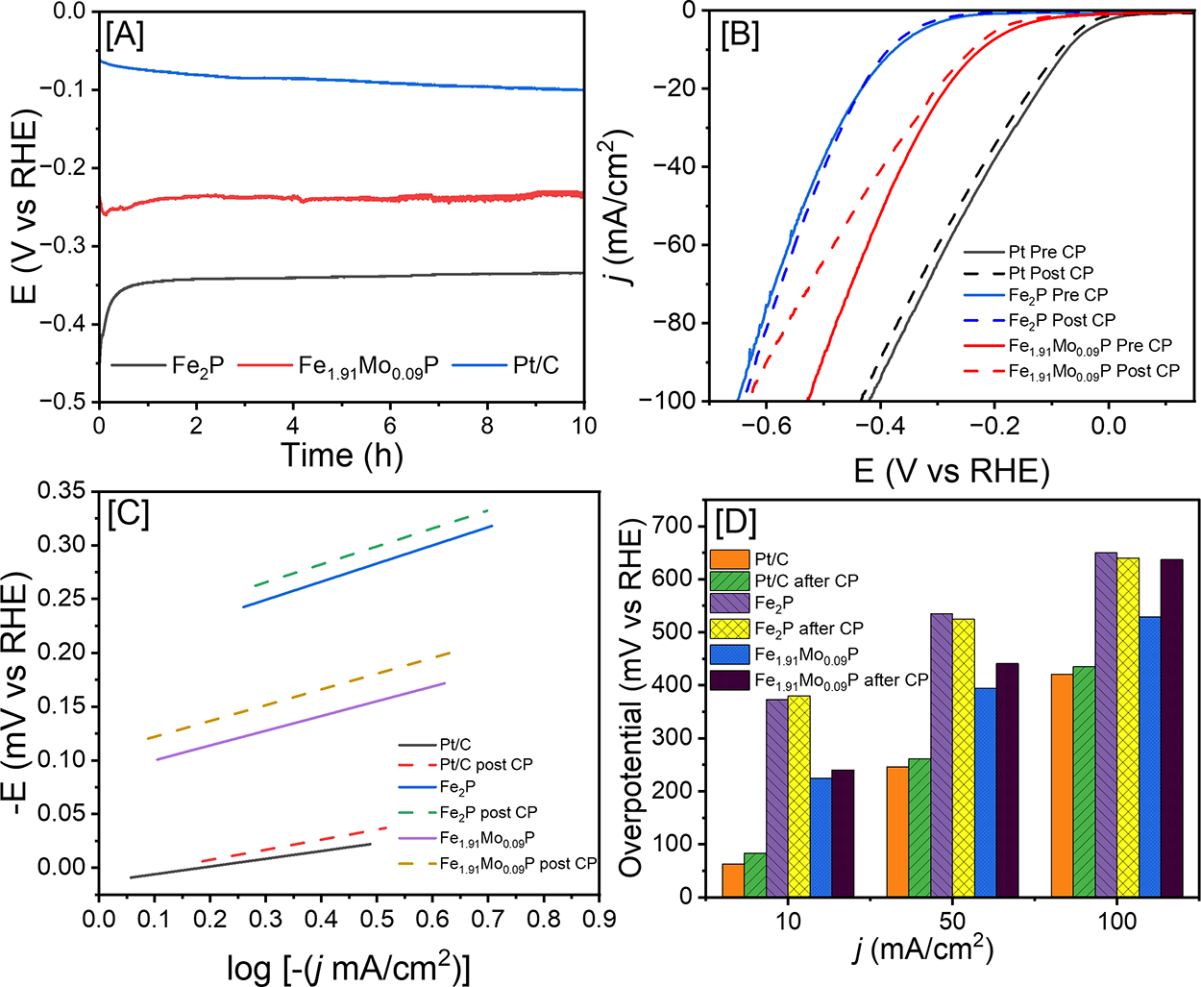
(A) Chronopotentiometry plot of Fe_2_P and Fe_1.91_Mo_0.09_P NRs at −10 mA/cm^2^ for 10 h. (B) LSV curves before and after CP for Pt/C, Fe_2_P, and Fe_1.91_Mo_0.09_P. Tafel slope calculations before and after CP for (C) Pt/C, Fe_2_P, and Fe_1.91_Mo_0.09_P. (D) Comparison of overpotential before and after CP for Pt/C, binary Fe_2_P, and Fe_1.91_Mo_0.09_P, respectively.

Chronopotentiometry was employed to investigate the effects of Mo doping on the stability of bimetallic NRs. Hexagonal Fe_2_P, the highest performing Fe_1.91_Mo_0.09_P NRs, and commercial Pt/C (10 wt %) were evaluated at constant *j* = −10 mA/cm^2^ in a N_2_-saturated 1 M KOH solution. Figure 7A demonstrates that Fe_2_P is stable over 10 h of continuous HER with a minimal increase of η_–10_ from 376 to 386 mV before and after chronopotentiometry, respectively, corresponding to a 2.7% increase (Figure 7B,D). The highest HER active Fe_1.91_Mo_0.09_P NRs also showed high stability in 1 M KOH with a minimal increase of η_–10_ from 225 to 240 mV before and after chronopotentiometry, respectively, corresponding to a 6.7% increase. Additionally, there was no presence of Mo leaching, as demonstrated by Table S3. The commercial Pt/C (10 wt %) standard showed an increase in η_–10_ from 61 to 83 mV, respectively, corresponding to a 36.1% increase. This data demonstrate that both binary Fe_2_P and Fe_1.91_Mo_0.09_P NRs show high stability in alkaline electrolytes, and the incorporation of Mo does not alter the stability of NRs significantly. Additionally, Tafel slopes were computed before and after chronopotentiometry to investigate the potential changes in HER kinetics. As shown in Figure 7C, Tafel slopes of 71.89 ± 0.66, 184.99 ± 0.30, and 167.55 ± 0.18 mV/dec were obtained for Pt/C (10 wt %), hexagonal Fe_2_P, and the highest HER active Fe_1.91_Mo_0.09_P NRs, respectively, before chronopotentiometry. After 10 h of HER, Pt/C displayed a 30.6% increase in Tafel slope (93.78 ± 0.84 mV/dec), thus indicating a notable decrease in reaction kinetics where commercial Pt became less efficient over time. Conversely, hexagonal Fe_2_P and the highest performing Fe_1.91_Mo_0.09_P NRs showed a minimal increase in the Tafel slope to 187.71 ± 0.24 and 170.28 ± 0.51 mV/dec, corresponding to 1.5% and 1.7% increases, respectively. This suggests that the rate kinetics are minimally affected by the incorporation of Mo, and the stability of mono- and bimetallic NRs was well maintained before and after the chronopotentiometry test. Additionally, this suggests that Fe_2–*x*_Mo_*x*_P NRs are robust catalysts that can be utilized over a longer period when compared with commercial Pt/C.

## Conclusions

In summary, the HER activity of hexagonal Fe_2_P and orthorhombic Fe_2–*x*_Mo_*x*_P NRs was investigated by using an integrated experimental and theoretical study. DFT calculations reveal that the orthorhombic Fe_2_P (210) surface displays a superior HER performance compared to the hexagonal Fe_2_P (111) surface. Dopants do not occupy the Fe triangle sites on the hexagonal Fe_2_P (111) surface, while dopants do occupy the Fe triangle on the orthorhombic (210) surface. This makes it easier to modify the surface electronic structure in the later system. Among eight dopants computationally studied for orthorhombic Fe_2_P, V, Co, Ti, and Mo exhibited superior HER activity compared to pristine Fe_2_P. Specifically, Mo doping was predicted to substantially decrease the overall |Δ*G*_H_| closer to a thermoneutral value. Accordingly, a series of Fe_2–*x*_Mo_*x*_P NRs with tunable compositions (x = 0.03–0.28) and hexagonal to orthorhombic structures were produced via colloidal synthesis. Structural analysis of Fe_2–*x*_Mo_*x*_P NRs showed a phase transition from hexagonal to orthorhombic Fe_2_P with increasing Mo concentration above 5.41% (*x* = 0.11). However, the original morphology of hexagonal Fe_2_P NRs was maintained with minimal changes in NR length and width for Fe_2–*x*_Mo_*x*_P NRs with *x* = 0–0.28 compositions. STEM-EDS elemental maps confirm the homogeneous solid solution behavior of Fe_2–*x*_Mo_*x*_P NRs produced. Surface analysis shows the presence of partially charged Fe^δ+^, Mo^δ+^, and P^δ-^ species and a change in surface chemistry with Mo incorporation. The mixed-phase Fe_2–*x*_Mo_*x*_P NRs showed higher HER activity compared to hexagonal Fe_2_P NRs. The lowest η_–10_ (222 mV) was achieved for Fe_1.91_Mo_0.09_P composition (4.58% Mo) consistent with the Δ*G*_H_ calculations, which predicted the optimum Δ*G*_H_ at 4.69% Mo as it exhibited the lowest overall |Δ*G*_H_| value of 0.26 eV. The highest performing Fe_1.91_Mo_0.09_P NRs showed the highest HER activity at all current densities (i.e., *j* = −10, −20, −50, and −100 mA/cm^2^) and a notable decrease in charge transfer resistance (*R*_ct_) upon homogeneous admixing of Mo at the optimal dopant concentration (*x* = 4.58%). Regardless of structure transition, all catalysts exhibit a Volmer–Heyrovsky HER mechanism with Tafel slopes ranging from 189.45 ± 0.57 to 278.33 ± 0.17 mV/dec. The lowest Tafel slope of 167.08 ± 0.23 mV/dec was achieved for the Fe_1.91_Mo_0.09_P NRs (4.58% Mo). Chronopotentiometry revealed that the incorporation of Mo had a negligible effect on the stability of the bimetallic catalysts. When compared to Pt/C, Fe_2_P and Fe_2–*x*_Mo_*x*_P NRs displayed superior stability and retained their original HER activity more effectively in alkaline electrolytes.

## Supporting Information Available

Computational figures of doping studies on hexagonal Fe_2_P (111) surface and the corresponding Δ*G*_H_ values; PXRD patterns of Fe_2_P and Fe_2–*x*_Mo_*x*_P with varying Mo compositions; STEM images and elemental maps of Fe_1.75_Mo_0.25_P and Fe_1.86_Mo_0.14_P NRs; XPS survey spectra for Fe_2_P and Fe_2–*x*_Mo_*x*_P NRs; XPS spectra for Fe_1.91_Mo_0.09_P NRs; PXRD patterns of pre- and postannealed Fe_2_P NRs; HER activity data of heteroatom-doped Fe_2_P samples from the literature; and ICP-OES data of 1 M KOH electrolyte solution after 10 h of continuous HER (PDF)
