# Supplementary material for: Dopant-Induced Hexagonal to Orthorhombic Phase Transition in Fe2–xMoxP Nanorods and Its Influence on the Electrocatalytic Hydrogen Evolution Reaction
Source: Chem Mater. 2025 Apr 28;37(9):3260–73. doi: 10.1021/acs.chemmater.4c03479 (PMC12079791; doi:10.1021/acs.chemmater.4c03479)
Supplement: Supplementary file 1 — cm4c03479_si_001.pdf [file cm4c03479_si_001.pdf]

Dopant-Induced Hexagonal to Orthorhombic Phase Transition in $Fe_{2-x}Mo_xP$ Nanorods and Its influence on the Electrocatalytic Hydrogen Evolution Reaction

*Jordon Baker, Danyang Wang, Md Kawsar Alam, Ka Un Lao, Indika U. Arachchige**

Department of Chemistry, Virginia Commonwealth University, Richmond, Virginia 23284-2006, United States.

Supporting Information

*Email: iuarachchige@vcu.edu

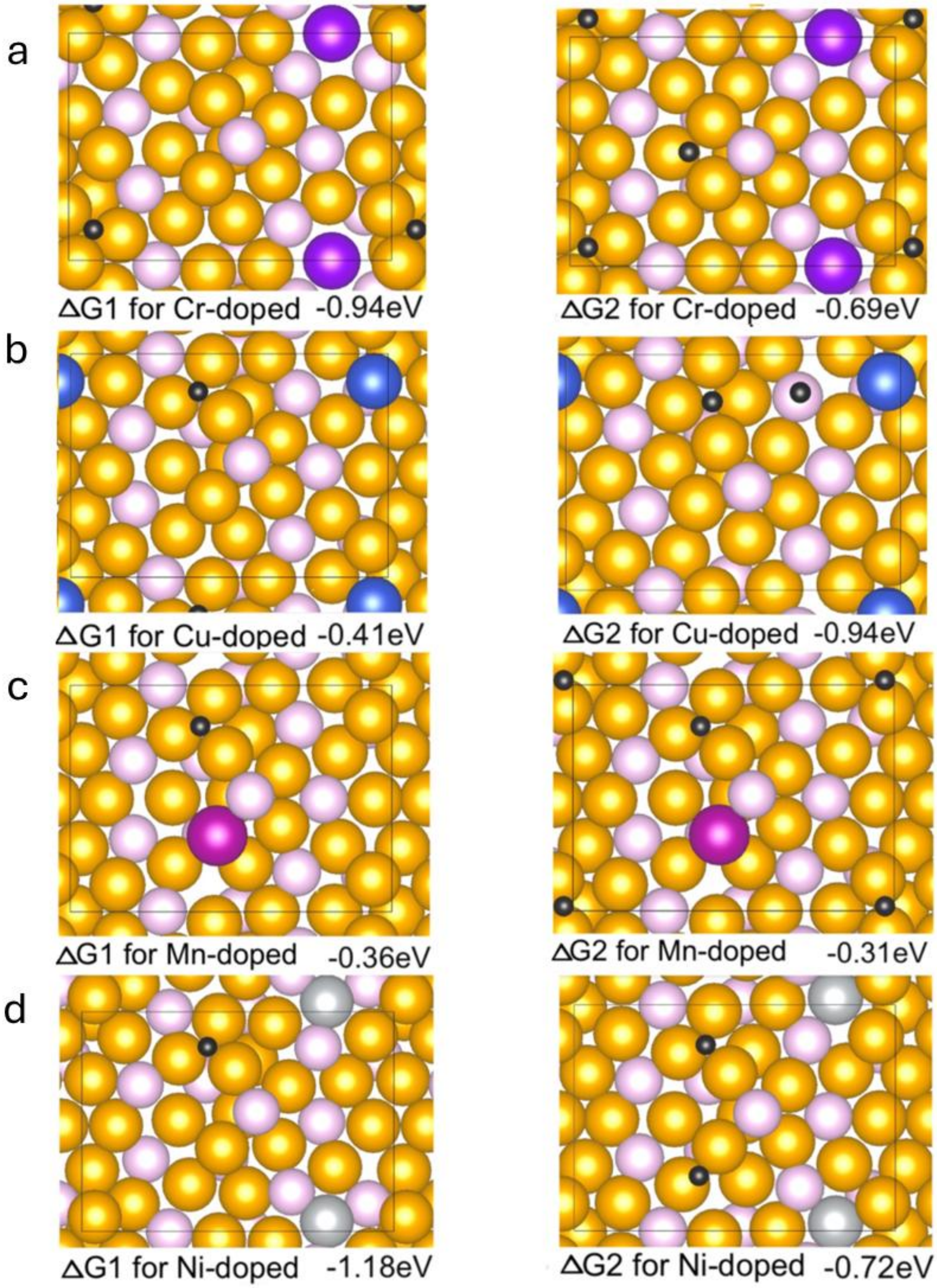

Figure S1. First and second ΔG_H on (a) Cr, (b) Cu, (c) Mn, and (d) Ni -doped orthorhombic Fe_2P (210) surfaces with $Fe_{11}P_7$ termination at a dopant concentration of 3.13 %. Fe atoms are shown in yellow, P atoms in pink, H atoms in black, and dopants in various colors.

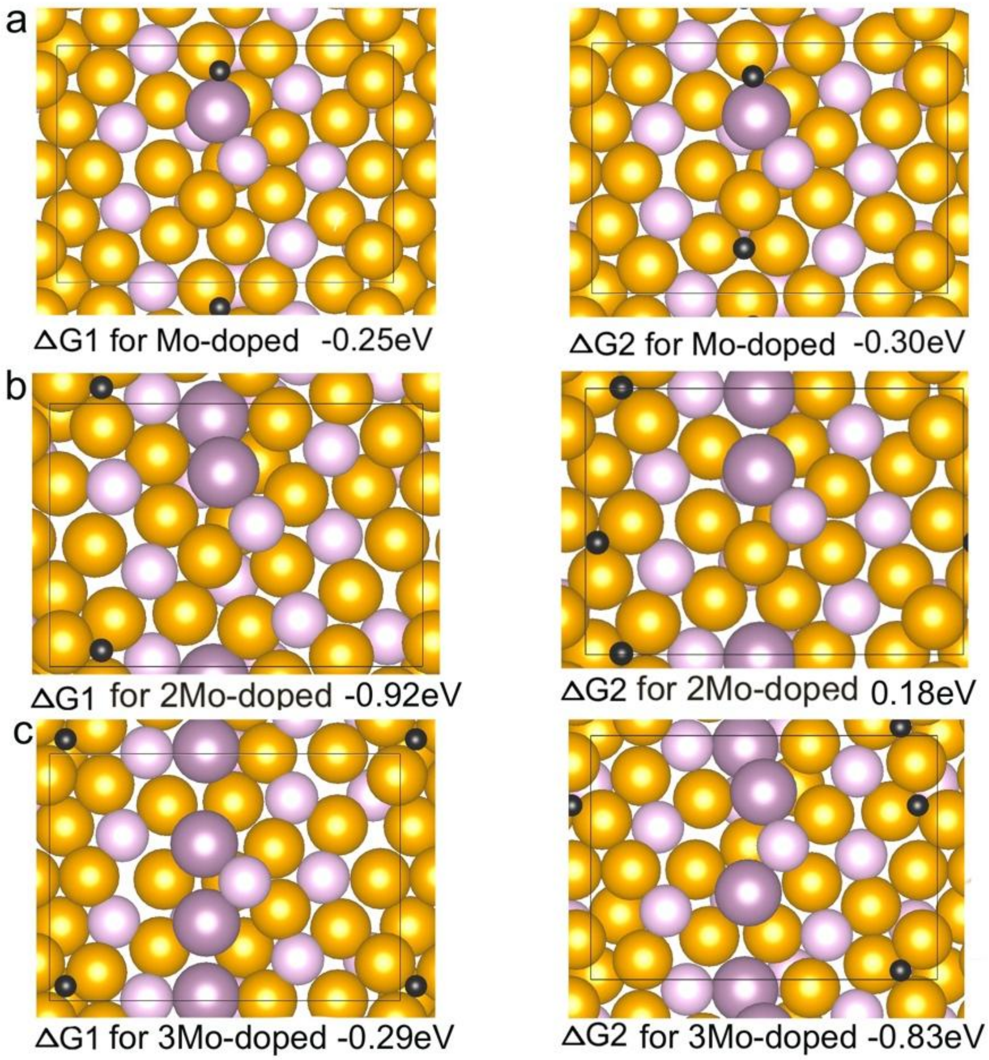

Figure S2. First and second ΔG_H on Mo-doped orthorhombic Fe_2P (210) surfaces with $Fe_{11}P_7$ termination at Mo concentrations of (a) 3.13, (b) 6.26, and (c) 9.39%. Fe atoms are shown in yellow, P atoms in pink, H atoms in black, and dopants in various colors.

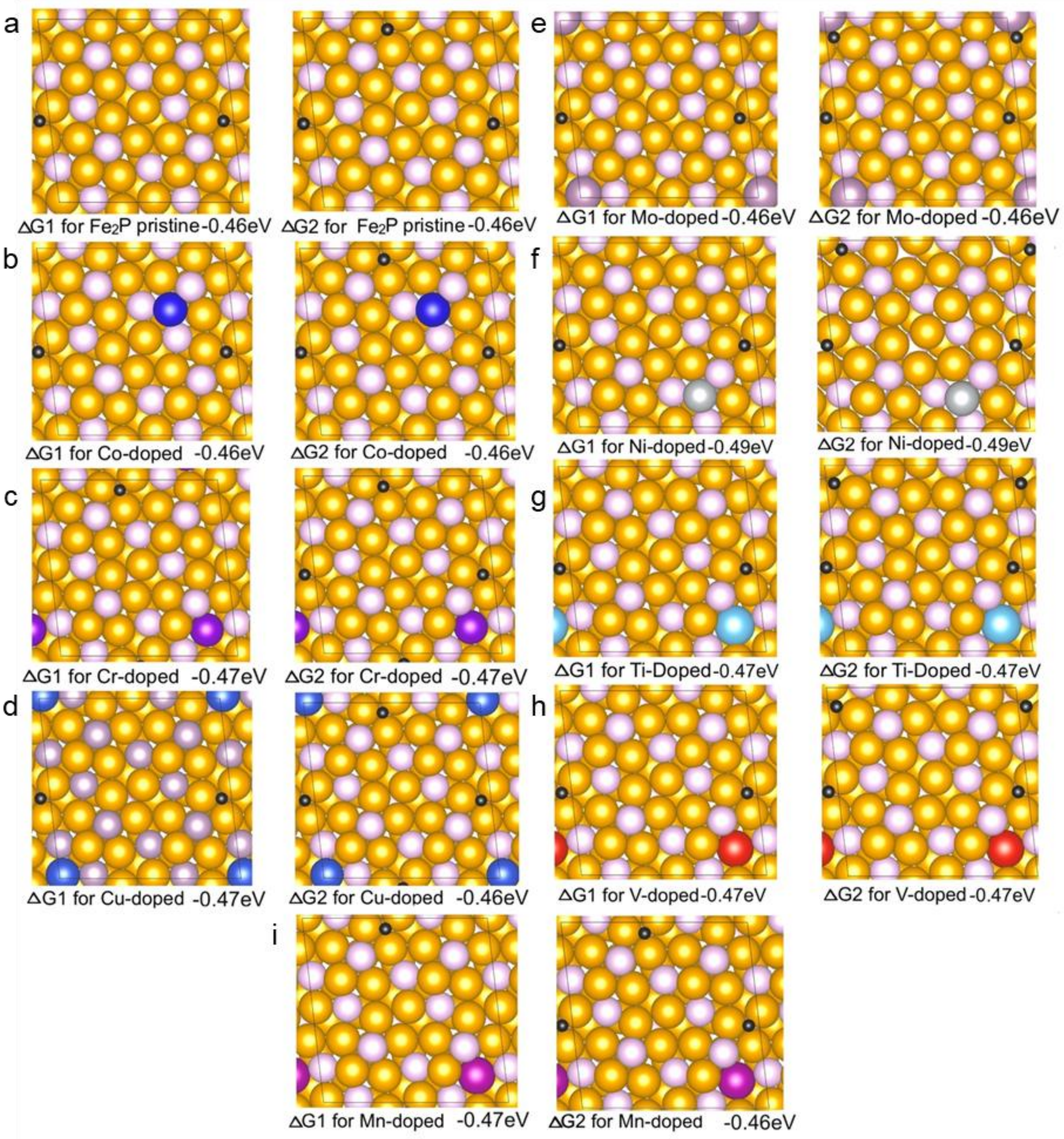

Figure S3. First and second ΔG_H values on (a) pristine Fe_2P (111) surface along with (b) Co, (c) Cr, (d) Cu, (e) Mo, (f) Ni, (g) Ti, (h) V, and (i) Mn-doped hexagonal Fe_2P (111) surfaces with $Fe_{24}P_{12}$ termination. Fe atoms are shown in yellow, P atoms in pink, H atoms in black, and dopants in various colors.

Table S1. First and second ΔG_H of pristine hexagonal Fe_2P (111) surface and hetero-atom-doped hexagonal Fe_2P (111) surfaces with $Fe_{24}P_{12}$ termination at a dopant concentration of 3.13%.

	ΔG_{H1} (eV)	ΔG_{H2} (eV)
pristine	-0.46	-0.46
Co	-0.46	-0.46
Cr	-0.47	-0.47
Cu	-0.47	-0.46
Mn	-0.47	-0.46
Mo	-0.46	-0.46
Ni	-0.49	-0.49
V	-0.47	-0.47
Ti	-0.47	-0.47

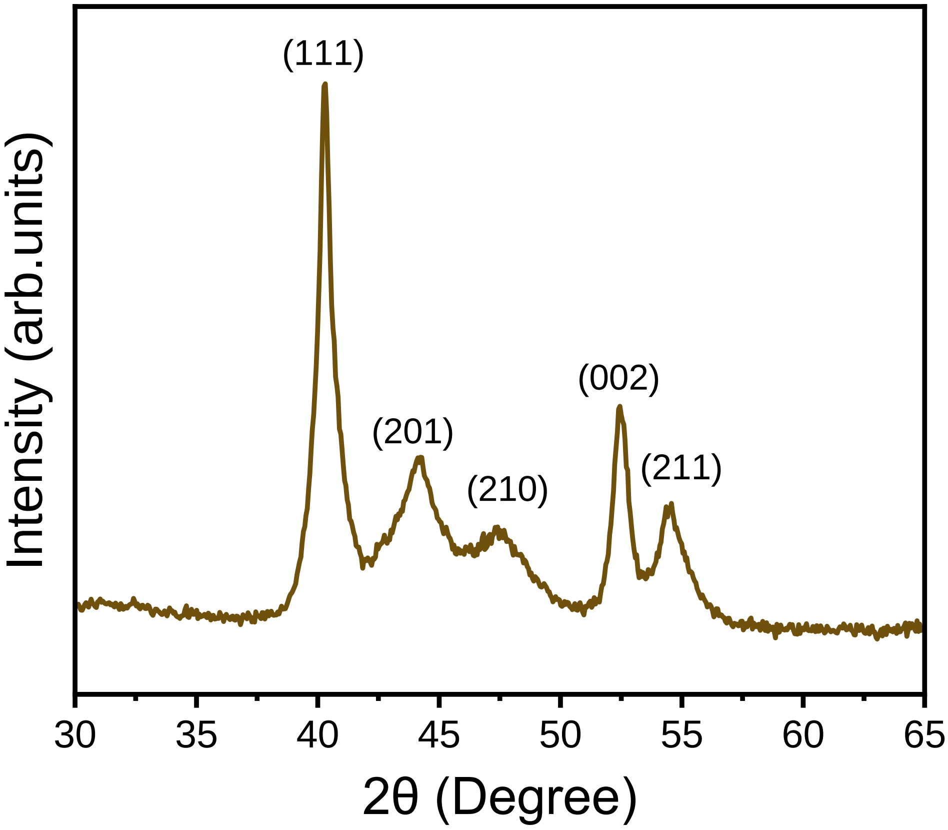

Figure S4. X-ray diffraction pattern of phase pure hexagonal Fe_2P NRs.

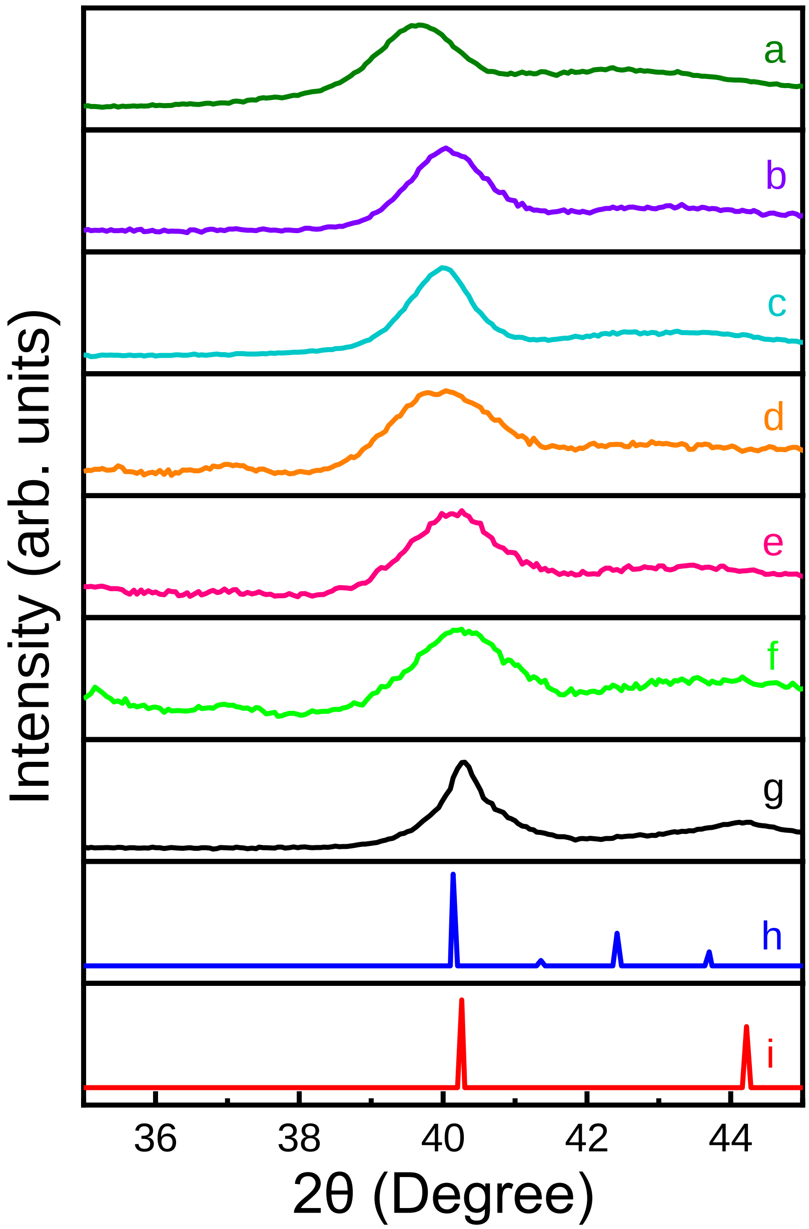

Figure S5. PXRD patterns of (a) $Fe_{1.76}Mo_{0.24}P$, (b) $Fe_{1.86}Mo_{0.14}P$, (c) $Fe_{1.89}Mo_{0.11}P$, (d) $Fe_{1.91}Mo_{0.09}P$, (e) $Fe_{1.94}Mo_{0.06}P$, (f) $Fe_{1.97}Mo_{0.03}P$, (g) Fe_2P NRs along with (h) orthorhombic Fe_2P (PDF 01-090-8789) and (i) hexagonal Fe_2P (PDF 01-078-6747) reference patterns.

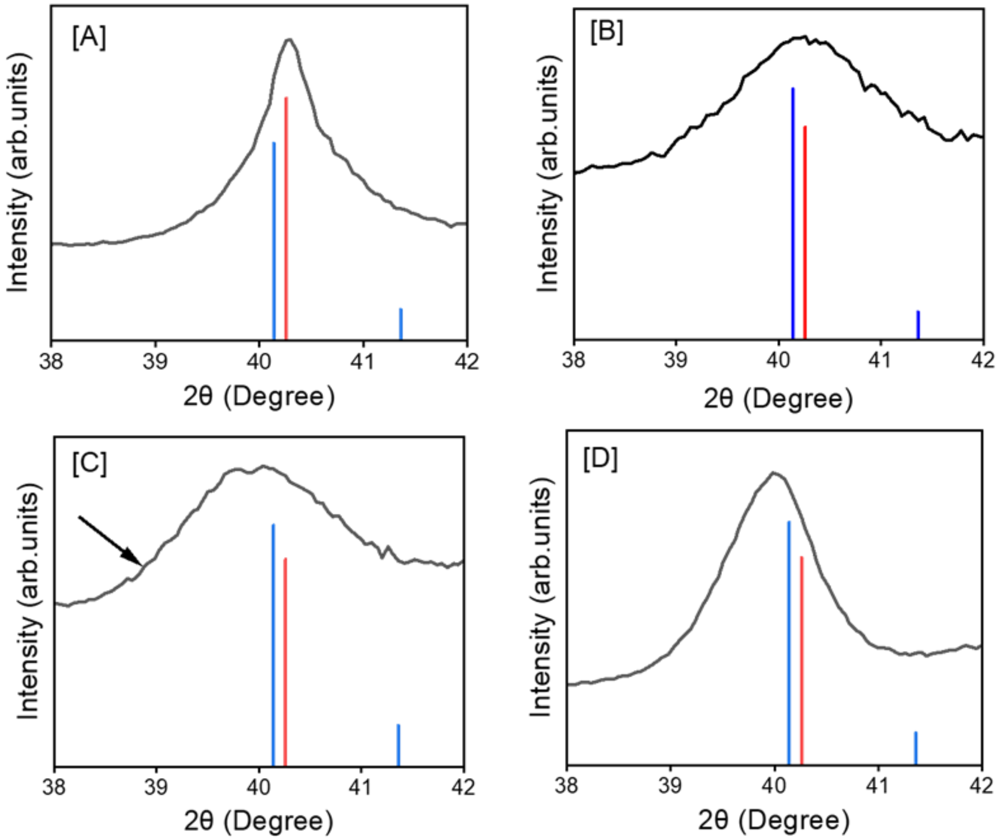

Figure S6. PXRD patterns of [A] Fe_2P, [B] $Fe_{1.97}Mo_{0.03}P$, [C] $Fe_{1.91}Mo_{0.09}P$, and [D] $Fe_{1.89}Mo_{0.11}P$ NRs displaying the 2θ = 38-42° region. All patterns are overlayed with the hexagonal (PDF 01-078-6747) and orthorhombic (PDF 01-090-8789) PDF reference patterns denoted in red and blue colors, respectively. Panel A shows phase pure Fe_2P NRs where the experimental pattern can be assigned to hexagonal Fe_2P with no asymmetry. However, panel B shows minor asymmetry (specifically towards lower 2θ values), which can be attributed to the combined effects of lattice expansion and structural transition from hexagonal to orthorhombic Fe_2P. Similarly, panel C shows clear asymmetry, including a pronounced left shoulder. This stems from a combination of the continued phase transition and expansion of the crystal lattice, resulting in a shift of the peak towards lower 2θ values. This asymmetry is not observed in panel D, which indicates that the phase transition had already taken place.

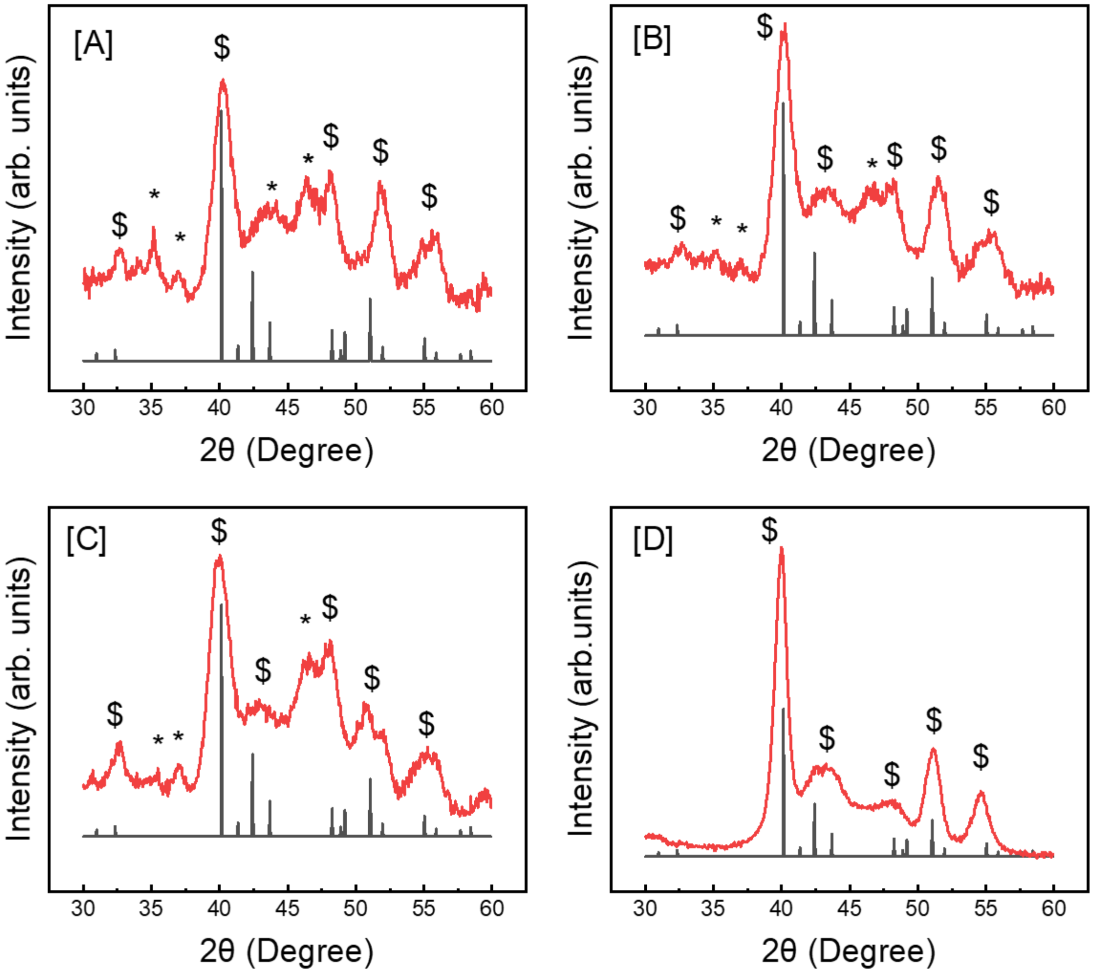

Figure S7. PXRD patterns of (A) $Fe_{1.97}Mo_{0.03}P$, (B) $Fe_{1.94}Mo_{0.06}P$, (C) $Fe_{1.91}Mo_{0.09}P$, and (D) $Fe_{1.89}Mo_{0.11}P$ NRs overlayed with orthorhombic Fe_2P (PDF 01-090-8789) reference pattern. The asterisk marks '*' denote peaks that can be assigned to hexagonal Fe_2P and the dollar signs '$' denote peaks that can be assigned to orthorhombic Fe_2P.

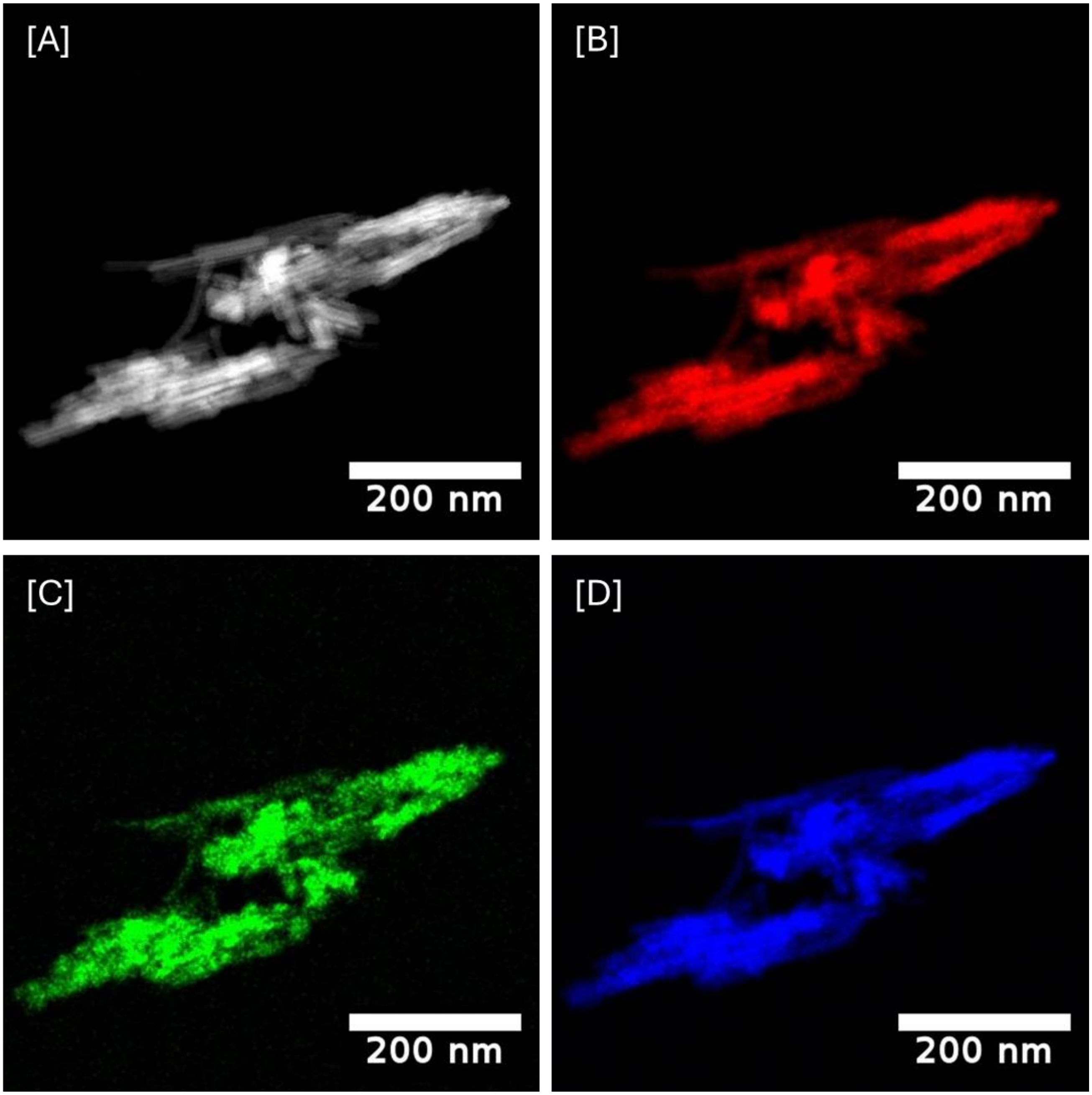

Figure S8. (A) A STEM image and STEM-EDS elemental maps of (B) Fe, (C) Mo, and (D) P recorded from $Fe_{1.75}Mo_{0.25}P$ NRs.

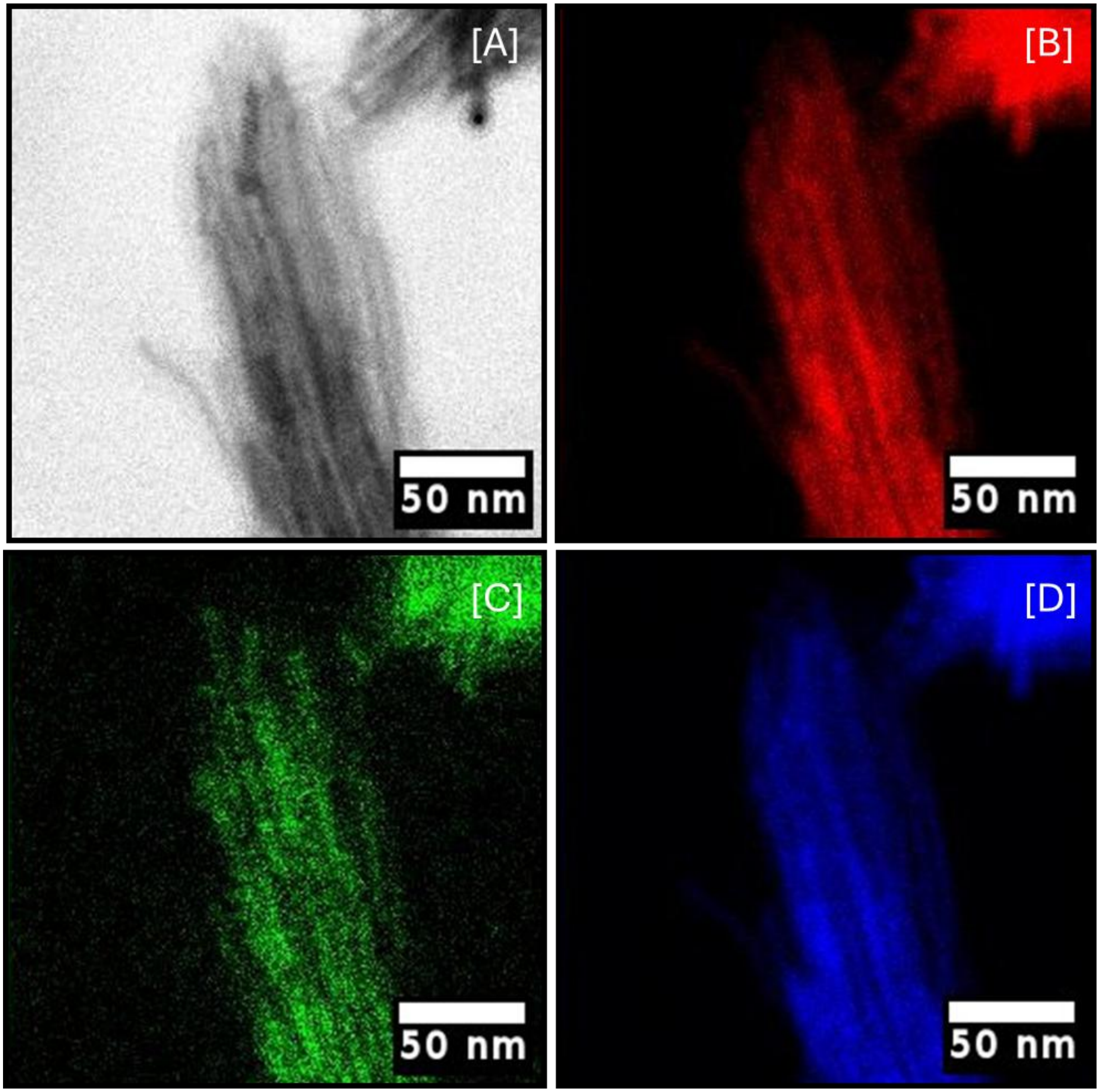

Figure S9. (A) A STEM image and STEM-EDS elemental maps of (B) Fe, (C) Mo, and (D) P recorded from $Fe_{1.86}Mo_{0.14}P$ NRs.

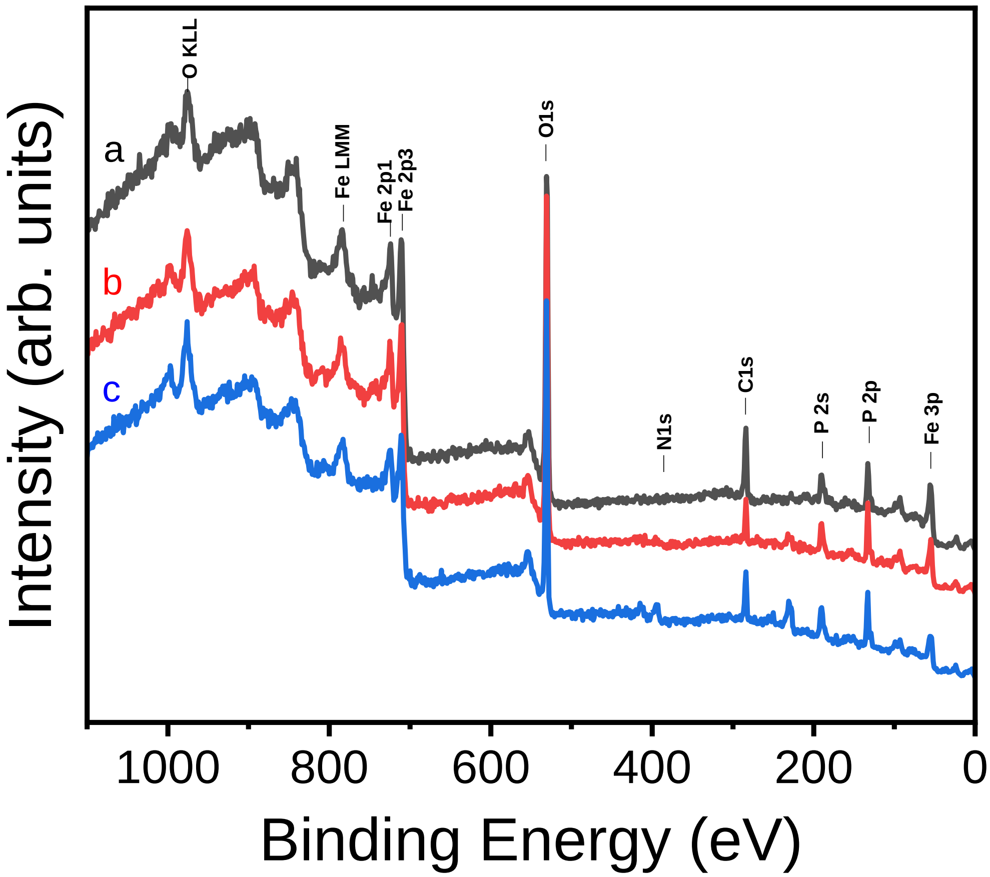

Figure S10. XPS survey spectra of (a) Fe_2P, (b) $Fe_{1.81}Mo_{0.19}P$, and (c) $Fe_{1.91}Mo_{0.09}P$ NRs.

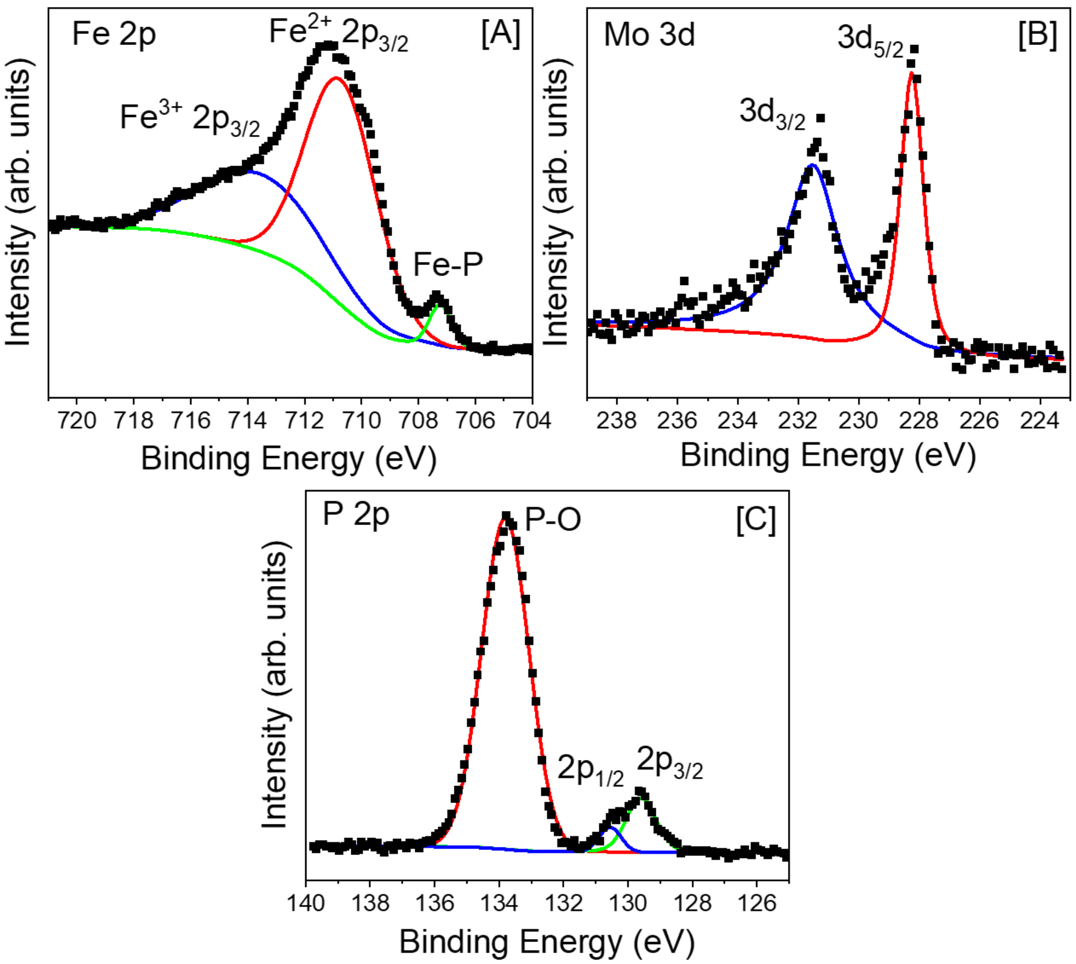

Figure S11. (A) Fe 2p, (B) Mo 3d, and (C) P 2p regional XPS spectra of $Fe_{1.91}Mo_{0.09}P$ NRs. Square symbols represent experimental data and colored lines are fitted deconvolutions. Samples were annealed for 2 h at 450 ºC under 5% H_2:Ar atmosphere.

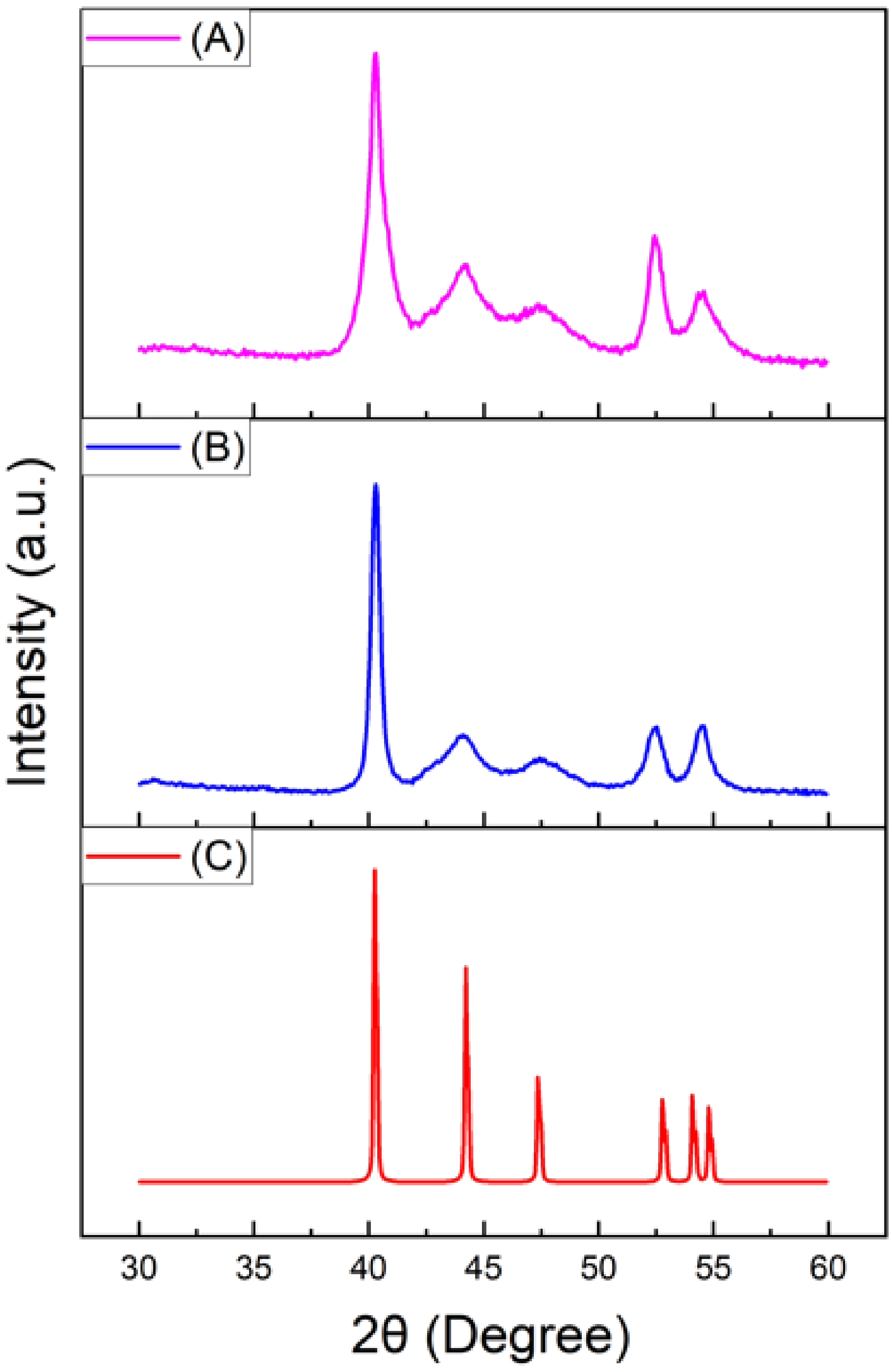

Figure S12. PXRD patterns of (A) as-synthesized Fe_2P NRs, (B) Fe_2P NRs annealed at 450 ºC for 2 h, along with (C) hexagonal Fe_2P reference pattern (PDF 01-078-6747).

Table S2. Literature reports of heteroatom-doped Fe_2P catalysts with variable morphologies deposited on different substrates for HER activity investigation. The corresponding HER overpotentials and Tafel slopes are also shown.

Sample	Substrate	Morphology	Acid/Base	η (mV)	Tafel Slope (mV/dec)	Reference
Fe_2P	Ti Foil	Nanorod	1M KOH	η_{-10}: 378	189.45 ± 0.57	
$Fe_{1.91}Mo_{0.09}P$	Ti Foil	Nanorod	1M KOH	η_{-10}: 222	167.08 ± 0.23	
Co-Fe_2P	Ni Foam	Nanofiber	Seawater	η_{-50}: 117	57	1
$Fe_{0.5}Ni_{1.5}P$	Ni Foam	Nanoparticle	0.5M H_2SO_4	η_{-50}: 163	65	2
V–Fe_2P@$Co_3(PO_4)_2$	Ni Foam	Nanosheets	1M KOH	η_{-10}: 56	39.05	3
Fe_2P–Ag@IF		Nanostructure	1M KOH	η_{-100}: 308.4	145.7	4

Table S3. ICP-OES Data of 1M KOH electrolyte solution after 10 h of continuous HER.

Element	Concentration (ppm)
Fe	0.04
Mo	0.01
P	0.01

References:

(1) Wang, S.; Yang, P.; Sun, X.; Xing, H.; Hu, J.; Chen, P.; Cui, Z.; Zhu, W.; Ma, Z. Synthesis of 3D Heterostructure Co-Doped Fe_2P Electrocatalyst for Overall Seawater Electrolysis. *Appl. Catal. B.* **2021**, *297*, 120386.

(2) Shin, D.; Kim, H. J.; Kim, M.; Shin, D.; Kim, H.; Song, H.; Choi, S.-I. $Fe_xNi_{2-x}P$ Alloy Nanocatalysts with Electron-Deficient Phosphorus Enhancing the Hydrogen Evolution Reaction in Acidic Media. *ACS Catal.* **2020**, *10*, 11665–11673.

(3) Liu, W.; Wang, L.; Gong, Y. Unique Ping-Pong Daisy-like Catalysts: Efficient Overall Hydrolysis Catalysis Driven by Vanadium-Doped Fe_2P and $Co_3(PO_4)_2$ Nanosheet Composites. *Int. J. Hydrogen Energy* **2024**, *69*, 401–410.

(4) Li, M.-X.; Zhou, Y.-N.; Dong, Y.-W.; Liu, X.; Luan, R.-N.; Liu, B.; Zeng, J.-B.; Chai, Y.-M.; Dong, B. Metal-Rich Heterostructure of Ag-Doped FeS/Fe_2P for Robust Hydrogen Evolution. *Int. J. Hydrogen Energy* **2022**, *47*, 20518–20528.
